# Multiscale fluorescence imaging of living samples

**DOI:** 10.1007/s00418-022-02147-4

**Published:** 2022-08-29

**Authors:** Yicong Wu, Hari Shroff

**Affiliations:** 1 Laboratory of High-Resolution Optical Imaging, National Institute of Biomedical Imaging and Bioengineering, National Institutes of Health, Bethesda, MD 20892 USA; 2 Present Address: Janelia Research Campus, Howard Hughes Medical Institute, Ashburn, VA 20147 USA

**Keywords:** Multiscale, Fluorescence, Microscopy, Live imaging

## Abstract

Fluorescence microscopy is a highly effective tool for interrogating biological structure and function, particularly when imaging across multiple spatiotemporal scales. Here we survey recent innovations and applications in the relatively understudied area of multiscale fluorescence imaging of living samples. We discuss fundamental challenges in live multiscale imaging and describe successful examples that highlight the power of this approach. We attempt to synthesize general strategies from these test cases, aiming to help accelerate progress in this exciting area.

## Introduction

**Fig. 1:**
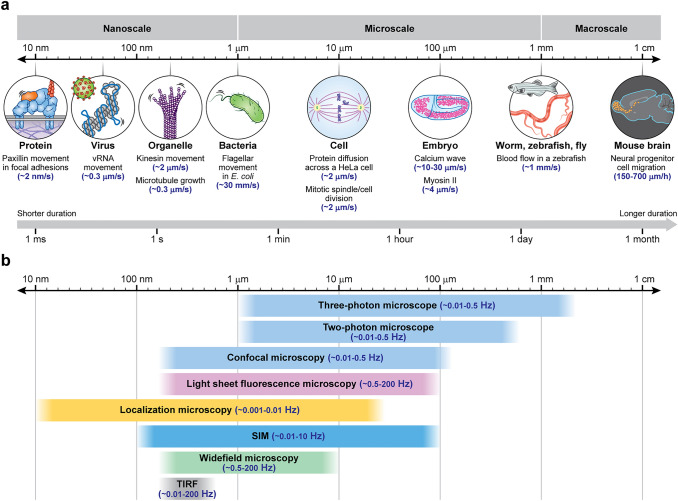
Multiscale imaging spans diverse spatiotemporal scales, which are usually accessed via distinct microscopy modalities. **a** Examples of biological phenomena that occur over the nanometer to centimeter spatial scale, and over timescales ranging from milliseconds to days. Note that rapid motion (here indicated by rates) occurs at all scales. **b** Typical fluorescence microscopy methods used in living samples over indicated spatiotemporal scales. Note that boundaries between methods are ‘fuzzy’ and only approximate

Movement is essential for life. Transcription factors diffuse within the nucleus, dynamically binding and unbinding stretches of nucleic acid to regulate gene activity. Molecular motors traverse intracellular distances, delivering valuable cargo precisely where and when it is needed. Cells divide, move, and communicate with their neighbors, building tissues which themselves dynamically interact to enable the development of embryos. Waves of calcium spreads within and across cells, generating activity patterns which result in organismal motion. These phenomena occur over the nanometer to meter spatial scale, and over timescales ranging from milliseconds to days (Fig. 1a).

As Yogi Berra famously said, ‘You can observe a lot by watching.’ Remarkably, modern fluorescence microscopy offers the ability to watch biological structure and function with molecular contrast and specificity, over the entire extent of these diverse spatiotemporal scales (Fig. 1b). The relatively recent field of super-resolution microscopy (Schermelleh et al. 2019) has improved spatial resolution to the point that nanoscale protein distributions can be visualized and tracked in living cells (Shroff et al. 2008; Balzarotti et al. 2017). Light-sheet microscopy (Power and Huisken 2017) now enables gentle, long-term imaging of cellular dynamics in transparent tissue (Keller et al. 2008; Wu et al. 2011), while multiphoton microscopy can enable high-resolution imaging of calcium activity even through an intact skull (Wang et al. 2018a).

In this review, we focus on multiscale imaging—the capability to traverse multiple spatial or temporal scales when performing a microscopy experiment. There has been exciting progress in this area, particularly on fixed samples. X-ray tomography can image entire model organisms (Ding et al. 2019), or even human organs (Walsh et al. 2021), with cellular resolution. Correlative workflows enable the ‘painting’ of organelles with super-resolution microscopy, within the full ultrastructural cellular context provided by electron microscopy (Hoffman et al. 2020; Kopek et al. 2012). Crossing even larger spatial scales, correlative methods can allow the same ~ cm^3^-sized mouse brain to be imaged in MRI and X-ray tomography, and then permit subvolumes of interest to be imaged at nanometer resolution with SEM (Foxley et al. 2021).

Performing multiscale fluorescence microscopy of living samples is the important yet understudied topic of this review. First, we discuss fundamental challenges in fluorescence microscopy, with an emphasis on those most pertinent for live multiscale imaging. Second, we describe successful ‘test cases’ that illustrate imaging across spatiotemporal scales using different microscopes. Finally, by studying these examples, we attempt to synthesize general strategies for those wishing to conduct their own multiscale imaging experiments on live samples.

## Basic considerations

**Fig. 2:**
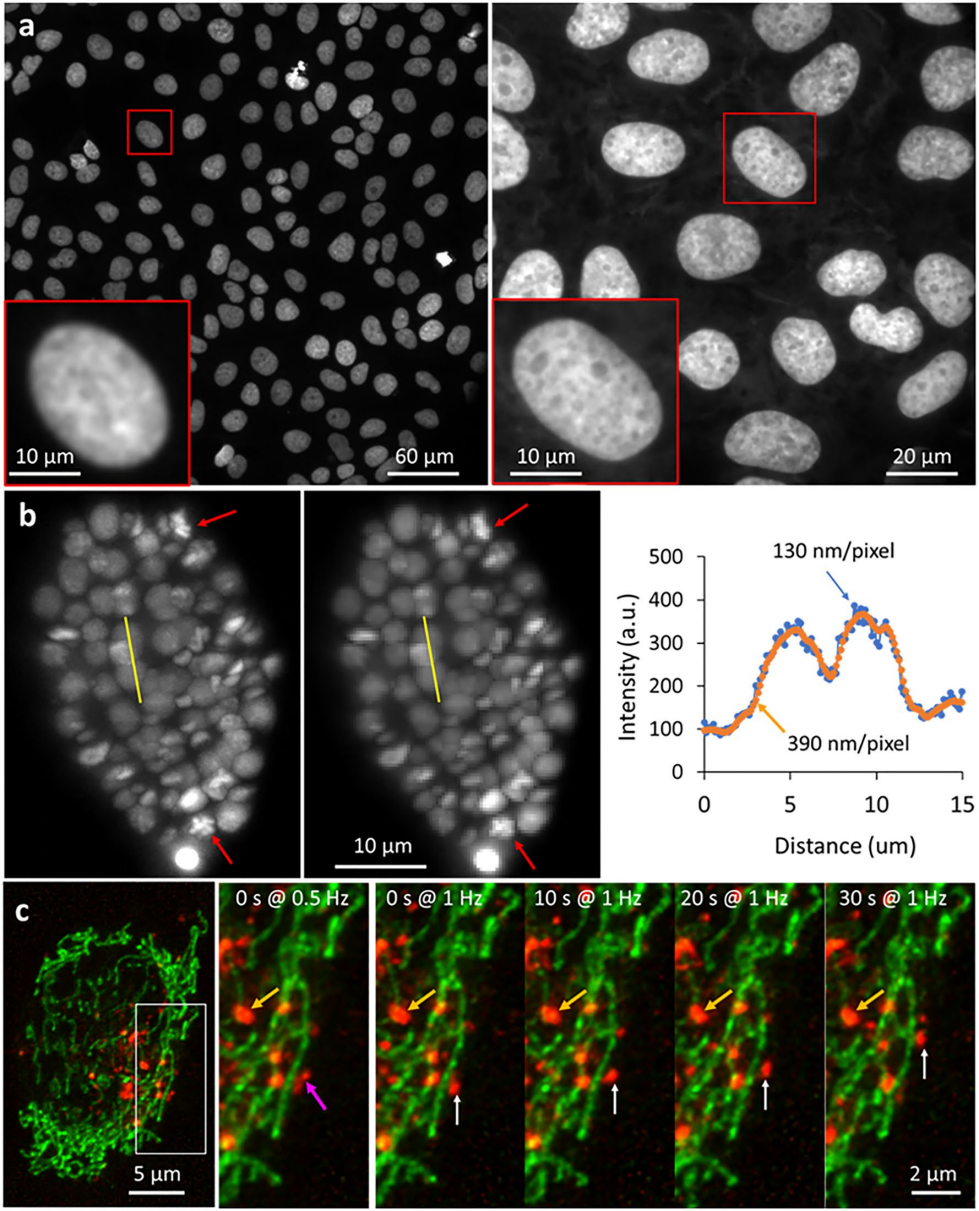
Basic considerations and tradeoffs in multiscale imaging. **a** Trading field of view (FOV) for spatial resolution. Images of DAPI-stained nuclei in fixed U2OS cells acquired with widefield microscopy.* Insets* show higher-magnification views of a single nucleus.* Left*: image acquired with 20x/0.5 NA dry objective lens;* Right*: image of a different field of view acquired with a 60x/1.42 NA oil immersion objective lens. A larger field of view (FOV) can be attained by using an objective with lower magnification, albeit with worsened spatial resolution. **b** Larger pixels compromise spatial resolution, but improve SNR. Images of GFP-histone-labeled *C. elegans* embryos acquired with light sheet microscopy (Wu et al. 2017).* Left*: with a pixel size of 130 nm;* Middle*: after digitally binning pixels to 390 nm to mimic the case when detector with larger pixels is used.* Right*: Intensity profiles over the yellow lines in images, showing improved SNR with larger pixel size. Larger pixels are sufficient to image coarser features (e.g., nuclei), but fine features within nuclei, evident during cell division (*red arrows*) are better resolved with a smaller pixel size. **c** Motion blur due to insufficient temporal resolution. Images of mitochondria (*green*) and lysosomes (*red*) in human colon carcinoma (HCT-116) cells, acquired with multiview confocal microcopy (Wu et al. 2021).* Left*: maximum intensity projection in lateral view at a typical time point;* Middle*: magnified view of the white rectangle in the most left column, acquired at rate of ~ 2 s per volume;* Right*: same as in the middle column, but acquired at rate of ~ 1 s per volume, showing four time points. Rapidly moving lysosomes (*magenta arrow*) are blurred at slower imaging rate, and better resolved when imaging at a faster rate (*white arrows*). Motion blur is less problematic at larger length scales (*orange arrows*)

Spatial resolution, field of view, signal-to-noise ratio (SNR), temporal resolution, and phototoxicity are intimately entwined in live imaging experiments. These experimental characteristics, and their implications for multiscale imaging, can be appreciated on a basic level by contemplating the pixels that constitute an image. Imaging at better spatial resolution implies sampling with smaller pixels (higher magnification), given the Nyquist–Shannon sampling theorem. For the same total number of detected pixels, a larger field of view (FOV) can be attained simply by using an imaging system with lower magnification, albeit with worsened spatial resolution (Fig. 2a). The practical consequence is that imaging optics well suited for high-resolution imaging—often high numerical aperture (NA), high magnification objective lenses—are ill suited for imaging larger FOVs, which are often better served by selecting lower-NA, lower-magnification optics. Commercial microscopes usually offer multiple lenses on an objective turret so that users can pick the lens best suited for a specific spatial scale. Although convenient, such systems are not designed with live multiscale imaging in mind, in which multiple spatial scales may need to be imaged in rapid sequence or simultaneously.

Pixel size also directly affects SNR, with smaller pixels collecting less signal than larger pixels. Attempting to resolve fine features with small pixels while maintaining acceptable SNR often requires a higher signal collection rate than imaging coarser features with larger pixels (Fig. 2b) (Winter and Shroff 2014). This challenge is compounded by label density, the amount of fluorescent probe that can be attached specifically to a target of interest. Small targets generally offer fewer sites for labeling, resulting in an additional lowering in SNR relative to larger structures. Resolving small, rapidly moving objects also necessitates faster imaging than required for resolving larger objects moving at the same rate, as the small object need only move by the resolution limit (i.e., spanning a few small pixels) during the image acquisition before introducing noticeable motion blur, but such blur may be negligible at larger length scales (Fig. 2c). Faster imaging in turn requires that the available signal be split into more temporal bins than slower imaging, further lowering SNR in each image. One consequence of these simple analyses is that imaging dynamic phenomena at smaller length scales is generally more difficult than imaging larger scale phenomena.

When traversing spatial or temporal scales, the total available signal budget is thus important to consider, particularly because this budget is usually finite in fluorescence microscopy. The signal budget in most live imaging experiments is practically limited by photobleaching, the irreversible destruction of the fluorescent tag. But even if bleaching is mild, in living samples it is usually a harbinger of the more general problem of phototoxicity, undesirable light-induced damage that can perturb the biology under investigation. The causes of phototoxicity and associated implications for the choice of microscope have been reviewed extensively elsewhere (Stelzer 2014; Wäldchen et al. 2015; Laissue et al. 2017). Here we simply note that introducing additional illumination—as might be required in live multiscale imaging—almost certainly implies more toxicity than imaging at a single length or timescale.

## ‘Conventional’ forms of fluorescence microscopy are best suited for interrogating distinct spatial scales

The myriad types of fluorescence microscopes can be classified according to the spatial scales they are best suited for (Fig. 1b). For studying biological dynamics in two dimensions within several hundred nanometers of the coverslip surface, total internal reflection fluorescence (TIRF) microscopy (Fish 2009) is an excellent choice. By restricting illumination to a thin zone of evanescently decaying energy, out-of-focus background is dramatically suppressed, leading to clear images with excellent SNR and contrast. The background rejection also makes TIRF an attractive choice when conducting super-resolution imaging studies (Li et al. 2015). A second advantage of confining the illumination to the vicinity of the coverslip is minimization of phototoxicity—thousands of images may be collected without obviously affecting the cell, making TIRF particularly well suited for live-cell microscopy. Widefield microscopy is usually the first technique applied to the next spatial scale, samples ranging in thickness from ~ 1–10 μm, i.e., the thickness of a typical eukaryotic cell. Unlike TIRF microscopy, in widefield microscopy volumetric images can be acquired by moving the sample with respect to the detection focal plane. Widefield microscopy, like TIRF microscopy, benefits from widefield detection: the entire focal plane is recorded at once. This makes it well suited for studying rapid biological dynamics. The weakness of widefield microscopy is its susceptibility to background, as widefield illumination illuminates the entire sample, and there is no rejection of out-of-focus emission light originating outside the focal plane. Deconvolution (Sarder and Nehorai 2006) can greatly improve contrast even in the presence of out-of-focus light, but fails if the fluctuating shot noise associated with the background swamps the in-focus signal within the focal plane.

Point-scanning confocal microscopy, in which a tightly focused illumination beam is scanned through a sample and the fluorescence filtered through a pinhole, excels in densely labeled, thick (~ 100 μm) samples that would otherwise defeat widefield microscopy. Like widefield microscopy, portions of the sample above and below the focal plane are illuminated during confocal imaging, generating out-of-focus fluorescence. Unlike widefield microscopy, a pinhole conjugate to the illumination focus physically blocks the great majority of out-of-focus emission from contaminating the image (which is collected on a point-detector such as a photomultiplier tube). This characteristic results in ‘optical sectioning (Conchello and Lichtman 2005)’, the isolation of in-focus signal from the out-of-focus background, a particularly useful attribute when performing 3D imaging. The superior background rejection of confocal microscopy comes at a price, however, which is a reduction in signal collection rate relative to widefield microscopy: the former collects signal serially, while the latter collects signal in parallel. The practical consequence is that, for a given frame rate, a widefield microscope usually collects much more signal than a confocal microscope. This is particularly salient when imaging large fields of view, when trying to image at very rapid frame rates, or when the SNR in point-scanning confocal microscopy may be limiting. For these applications, ‘hybrid’ spinning-disk, swept-field, or line-scanning confocal implementations that trade signal collection rate (better SNR and speed) for background rejection (Winter and Shroff 2014) may be better choices than the traditional point-scanning confocal microscope.

Both widefield and confocal microscopes illuminate the sample volumetrically, leading eventually to pronounced photobleaching and photodamage, which limit the duration of live imaging experiments. One of the most exciting developments in recent years is the advent of modern light-sheet fluorescence microscopy [LSFM (Power and Huisken 2017)], which confines the illumination to the vicinity of the focal plane and detects the resulting fluorescence perpendicularly to the illumination. Since illumination outside the detection plane is minimized, photodamage and photobleaching are drastically reduced relative to other forms of fluorescence microscopy (except TIRF microscopy, which uses even less illumination). Indeed, in marked contrast to confocal imaging, multiple LSFM studies (Wu et al. 2011; McDole et al. 2018) have reported a rate of photobleaching so low that it is outmatched by the rate of fluorescent protein synthesis, causing fluorescence to actually increase over time. The potent combination of optical sectioning, rapid imaging rates, high resolution, and low photodamage make LSFM a superb choice for live multiscale imaging from the subcellular to tissue level (Wu et al. 2013b; Chen et al. 2014). Several caveats are nevertheless still worth noting. First, in highly scattering, thick samples, confocal microscopy still holds an advantage over LSFM, in that the combination of highly focused illumination with confocal pinhole better rejects background than LSFM. Second, although spatial resolution continues to improve in LSFM, the highest NA implementations (Yang et al. 2019; Sapoznik et al. 2020) sacrifice valuable signal to improve lateral resolution. Finally, unlike commercially available confocal systems, most LSFMs are designed with a single magnification level in mind, implying that a particular LSFM implementation is best suited for a specific spatial scale.

## Imaging at small and large length scales

Imaging biology at very small or large length scales presents unique challenges. At small scales, the optical diffraction limit of ~ 250 nm hindered the interrogation of subdiffractive phenomena until relatively recently. The advent of super-resolution optical microscopy heralded a new era, in which nanoscale imaging, even of living systems, became possible in principle. In practice, any improvement in spatial resolution carries a hefty price (Ji et al. 2008; Winter and Shroff 2014)—either in increased acquisition time, increased photodamage, decreased penetration depth, or decreased SNR relative to diffraction-limited microscopy. In general, the more ‘extreme’ the gain in resolution, the higher the price that must be paid. Although this conclusion follows from the pixel-based arguments above, it is also worth remembering that the different implementations of super-resolution microscopy come with their own unique advantages and drawbacks (Schermelleh et al. 2019).

To appreciate how the choice of imaging modality relates to imaging scale, we find it useful to classify super-resolution techniques into three main types: single-molecule imaging methods, STED microscopy, and structured illumination microscopy. Single-molecule imaging methods (Lelek et al. 2021) [SMI, e.g., (fluorescence) photoactivated localization microscopy ((f)PALM (Betzig et al. 2006; Hess et al. 2006)); stochastic optical reconstruction microscopy (STORM (Rust et al. 2006)) and their many derivatives] cycle the emission of fluorophores between bright and dark states, isolating the fluorescence from a sparse subset of well-separated molecules and localizing the emission from each one. Since the fluorescence from a single molecule may be localized to nanometer precision, well below the diffraction limit, repeating the process of isolation and localization results in a super-resolution image. While SMI offer the best resolution (~ tens of nanometers in practice) of all techniques, the repeated cycling between dark and bright molecular states and the need to accumulate the localizations from tens to hundreds of thousands of molecules typically entails far higher illumination dose than diffraction-limited microscopy—limiting the total duration and speed of experiments. A second challenge is that single-molecule fluorescence is relatively weak, easily obscured by background. For these reasons, most live SMI experiments have been limited in spatial scale to single cells, and to temporal scales of ~ tens of seconds.

Stimulated emission depletion (STED) microscopy (Vicidomini et al. 2018) excites molecules within a diffraction-limited focus, forcing all emitters, except those in a nanoscale region to undergo stimulated emission rather than fluorescence. Scanning the nanoscale region through the sample produces a super-resolution image. In practice, STED microscopy offers resolution in the 50 to 150-nm range, somewhat worse than SMI but still considerably better than diffraction-limited microscopy. One advantage of STED microscopy is that it is built upon a confocal microscope and is thus more robust to background due to pinhole-based rejection of out-of-focus light. This characteristic allows STED imaging to be performed in tissue (Berning et al. 2012) as well as single cells. Similar to confocal microscopy, the scanning nature of STED implies that rapid rates can be attained over a very small FOV (Westphal et al. 2008), but that imaging larger FOVs take proportionately more time (Nägerl et al. 2008). Two major challenges when attempting live STED microscopy are photobleaching/phototoxicity (high STED intensities are required to outcompete fluorescence) and low signal generation (since much less fluorescence is generated per scan position than in confocal microscopy).

In structured illumination microscopy [SIM (Wu and Shroff 2018)], sharp illumination patterns are used to excite fluorescence from diffraction-limited (or smaller) regions of the sample, creating higher-resolution information that is encoded in the raw images captured by a detector. Decoding the images mathematically or optically yields a super-resolution image. Although the spatial resolution improvement offered in most forms of SIM is only twofold better than diffraction-limited microscopy, and thus considerably lower than in STED microscopy or SMI, SIM offers other advantages for multiscale imaging. First, the low illumination intensities in SIM make it the only technique capable of volumetric time-lapse imaging (‘4D’ imaging) over hundreds of time points, enabling sustained super-resolution imaging over many minutes (Shao et al. 2011). Second, although traditional implementations of SIM are susceptible to high levels of background, limiting application to single cells, newer implementations combine the resolution enhancement of SIM with the background rejection capability of confocal microscopy, enabling super-resolution imaging in tissue (York et al. 2012, 2013; De Luca et al. 2013). Finally, SIM is also the most rapid super-resolution method, enabling video-rate or faster (York et al. 2013; Guo et al. 2018) imaging of living processes.

Moving from smaller to larger scales, a different set of problems present themselves. For samples thicker than a single cell, refractive index variations within the sample lead to undesirable bending of the illumination and emission light. Such ‘optical aberrations’ often manifest as the inability to produce a diffraction-limited focus, leading to reduced contrast, SNR, and resolution in images. Another example of a very common, yet often ignored, aberration is the ‘focal shift (Bratton and Shaevitz 2015; Hell et al. 1993)’ that results when the refractive index of a sample differs from the refractive index of the immersion media that the objective lens is designed for. In this case, when acquiring volumetric data, the apparent axial distance between focal planes appears different than the true axial shift, distorting the axial extent of objects. Although this situation can be corrected in post-processing (once the user is aware there is a problem!), the best practice is to select an objective lens designed to image into a refractive index similar to the biological sample’s refractive index (realizing that one can only match the ‘average’ refractive index of the specimen). In other words, for imaging living samples, particularly at distances far from the coverslip, a water or silicone oil objective is preferred compared to an oil immersion lens.

A second common aberration encountered when performing fluorescence microscopy of 3D samples is spherical aberration. This aberration arises because high-angle illumination and emission rays are bent differently than low-angle rays, resulting in ‘smearing’ of the point spread function (PSF) along the optical axis. The consequence of this distorted focus is a significant loss in axial resolution and signal intensity in the focal plane. Most higher NA objectives contain movable optical elements (accessed via the ‘correction collar’) that can compensate for minor spherical aberration, but only at a fixed focal plane. In principle, adjusting the correction collar for each imaging depth could allow partial depth-dependent correction, but this strategy is slow (limited by mechanical movement of the collar) and fails to compensate for other sample-induced aberrations.

‘Adaptive optics’ [AO (Ji 2017)] refers to a family of techniques designed to redress optical aberrations. A complete description of these methods falls outside the scope of this review, here we simply recognize that any AO method: (1) must measure the aberration, or more precisely sense the aberrated ‘wavefront’ of the illumination and/or the emitted fluorescence; (2) must then apply a corrective signal to cancel the aberration, e.g., with a deformable mirror or spatial light modulator. The ‘sensing’ part of this loop is currently an active area of research, as there is no consensus about the best approach. We further note that incorporating AO into fluorescence microscopy is not trivial, and is thus currently limited to a relatively small number of labs that possess the technical skill to build their own AO subsystem and program the AO correction loop. Despite these difficulties, the payoff in spatial resolution, contrast, or SNR when implementing AO correction is potentially high, particularly in large, heterogenous samples with slowly varying changes in refractive index. AO holds particular promise for multiscale imaging, as it has proven essential for discerning subcellular (Wang et al. 2015) [or subdiffractive (Zheng et al. 2017)] structure deep in aberrating samples.

AO correction is most useful in extending the effective depth penetration of fluorescence microscopy in transparent tissues in which the aberration varies slowly laterally or axially. In highly scattering tissue, the aberration may vary rapidly, i.e., on a length scale too small to easily correct. In highly absorptive tissue, attenuation of the illumination light is the limiting factor. Scattering and absorption together result in an exponential loss in ‘ballistic’ illumination photons that create useful signal, as opposed to background. Since scattering decreases at higher wavelengths, and absorption of water and tissue is minimal from visible to infrared wavelengths, using near-infrared (NIR) illumination to excite redder dyes can help to extend the imaging depth (with the caveat that NIR-excitable dyes are typically less photostable and bright than those excited in the visible). Multiphoton (both two (Denk et al. 1990)-photon and more recently three (Horton et al. 2013)-photon) microscopy (Hoover and Squier 2013) offers another route to deeper imaging as (1) it is possible to efficiently excite many bright fluorophores with single-photon absorption spectra in the visible; (2) the longer illumination wavelengths are less scattered than single photon illumination; (3) even if illumination-side scattering occurs, it is far less likely than single-photon microscopy to drive associated background fluorescence due to the nonlinear intensity dependence of multiphoton microscopy; (4) up to a limit, scattered fluorescence may be collected by a ‘non-descanned’ detector and usefully assigned to the illumination focus. By increasing the laser power to compensate for the exponential loss in signal, these methods have enabled imaging millimeters into scattering tissue—a remarkable achievement given that single-photon confocal or light-sheet imaging generally fails within a few hundred microns of the sample surface. Ultimately, however, the benefits accrued by increasing illumination intensity are defeated by photodamage (Hopt and Neher 2001) (in a sample-dependent manner) or background [fluorescence generated at more superficial layers overwhelms the signal at the illumination focus (Theer and Denk 2006)].

When attempting imaging deep into living animals, it is worth considering difficulties due to the physiology of the live animal itself. Breathing, eye movement, heartbeat, or simply global animal motion are additional considerations that confound multiscale imaging, as they make it difficult to attain high-resolution imaging due to motion blur. Effective strategies to combat these issues include anesthetizing the animal (keeping in mind the potential effects on the biology under study), immobilizing part of the animal [as in ‘head fixed’ brain-imaging experiments (Schwarz et al. 2010)] or imaging rapidly enough that motion blur is not a concern (which may be difficult depending on the imaging modality and finite photon budget).

## Recent examples of successful multiscale imaging

### Combining distinct microscopy modalities

**Fig. 3:**
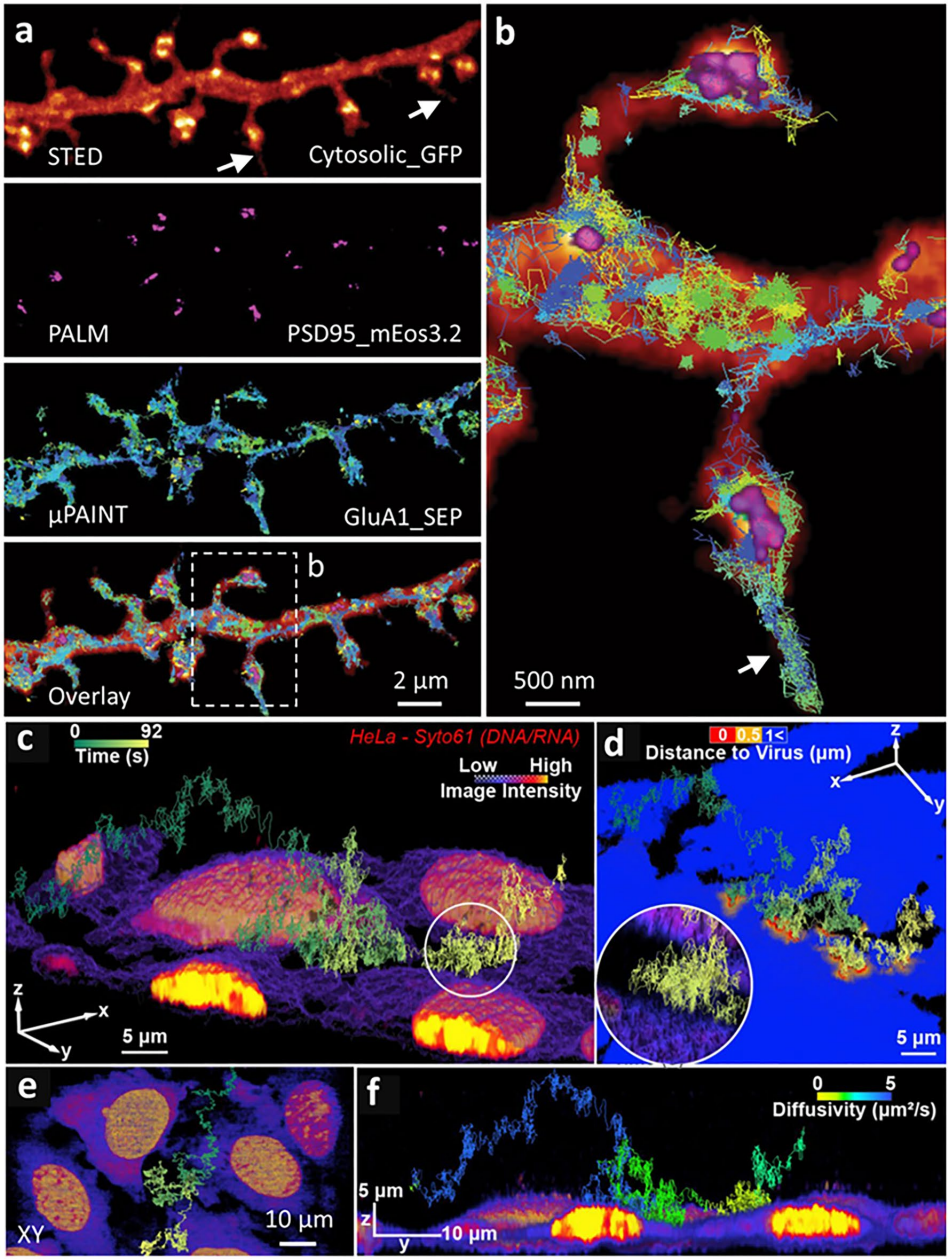
Multiscale imaging by combining distinct microscopy modalities. **a** STED, PALM, and µPAINT enable imaging of molecular trajectories within the context of super-resolved dendritic spines in a living hippocampal neuron. **b** Magnified view of two spines from the overlay image in (**a**), showing GluA1_SEP AMPA receptor trajectories inside and outside the PSD95 area, as well as freely moving along dendrites and spinules (*white arrows*). Individual trajectories are shown as distinct colors. **c**, **d** 3D reconstruction of a single vesicular stomatitis virus (VSV)-G pseudotyped lentiviral vector particle trajectory, as it ‘skims’ along the surface of live HeLa cells. The particle trajectory and cell morphology were simultaneously interrogated by combining 3D tracking and imaging (3D-TrIm), integrating real-time active-feedback tracking microscopy with a volumetric imaging system. Cells are color-coded by intensity and distance from the cell surface to the virus trajectory in (**c**) and (**d**), respectively.* Circular inset*: enlarged view of “skimming” event. **e** XY view with trajectory superimposed. **f** YZ view of the same cell and trajectory, with trajectory color-coded according to diffusion coefficient. **a**, **b** were reprinted with permission from ref (Krishna Inavalli et al. 2019), and **c**–**f** reprinted with permission from ref (Johnson et al. 2021)

Given the difficulties outlined above in ‘seeing everything’ with a single microscopy modality, combining multiple optical techniques in a single experiment (Hobson and Aaron 2022) is an effective choice for multiscale imaging. Such correlative methods can fill the gaps from the nanoscale (e.g., protein localization), to microscale (e.g., morphology of organelles/cells) to macro- or large-scale (e.g., groupings of cells in tissues and organs) structure of biological samples, while also permitting inspection of dynamics over different timescales. For example, a recent study merged multiple super-resolution imaging methods on a single microscope platform for multiscale imaging of dynamics in living neurons (Krishna Inavalli et al. 2019). Combining STED microscopy and its related super-resolution ‘shadow imaging’ [SUSHI (Tønnesen et al. 2018)] variant with single-particle tracking photoactivated localization microscopy [sptPALM (Manley et al. 2008)] and universal point accumulation imaging in nanoscale topography (Sharonov and Hochstrasser 2006) [uPAINT (Giannone et al. 2010)], enabled analyses of nanoscale synaptic protein dynamics in the context of dynamic neuronal morphology, including growth cones and dendritic spines. Using this multimodal platform, it was observed that PSD95 molecules (imaged via PALM) formed clusters mostly localized within spine heads in live hippocampal primary neurons expressing cytosolic GFP (imaged via STED), whereas AMPA receptor subunit GluA1 (imaged via uPAINT) diffused widely across the dendrite and stabilized at synapses (Fig. 3a, b). Furthermore, the spatial organization and mobility of glutamate receptors in different subcellular synaptic compartments, including dendrite, spine neck, spine head and spinules, were characterized by measuring the diffusion coefficient (which varied from 10^−4^ to 1 μm^2^ s^−1^ over different uPAINT trajectories). In addition, by rapidly switching between SUSHI imaging of a fluorescent marker (calcein) outside of the cells and sptPALM of mEos3.2-tagged actin molecules, retrograde actin flow along individual filopodia emanating from growth cones could be resolved with better than 100-nm spatial resolution. Such a process is very challenging to image with conventional super-resolution techniques due to the phototoxicity and low signal associated with intracellular labeling and imaging these fine structures.

A second multi-modal approach combines high speed 3D tracking of single viruses with contextual 3D cellular imaging [3D-TrIm (Johnson et al. 2021)] to capture the early stages of virus–cell interactions. Simultaneous study of both viruses and the surrounding cellular environment have been challenging due to the rapid, ms-scale diffusion of virions in the extracellular space and the comparatively large 3D cellular structures involved. For example, although a single moving virion can be localized with a precision of ~ 20 nm laterally and ~ 80 nm axially, the surrounding 3D environment can span tens of microns in each dimension. While interrogating such disparate scales using a single rapid volumetric scan might be possible, the authors instead chose to adopt distinct strategies well suited for each spatiotemporal scale by combining real-time active feedback tracking (for following viruses) with a volumetric imaging system (to obtain the surrounding context). The single-particle tracking system used electro-optic deflectors (EOD) and a tunable acoustic gradient (TAG) lens to rapidly scan and ‘lock on’ to the location of the fluorescently labeled virus, using stage feedback to keep the virus centered in the illumination focus. At the same time, the imaging system used a two-photon laser scanning microscope outfitted with an electrically tunable lens (ETL) to capture the cellular context in the vicinity of the particle. The combined imaging system allowed simultaneous interrogation of each vesicular stomatitis virus (VSV)-G pseudotyped lentiviral vector particle trajectory and its surrounding live-cell environment spanning ~ 100 × 100 × 25 μm^3^ (Fig. 3c–f). Virion positions were recorded at the ms timescale for ~ 90 s over an axial distance extending more than 20 μm above the cell surface. This rapid and large-range recording enabled the measurement of viral diffusion ranging from 0.5 to 4.5 μm^2^/s, also revealing ‘skimming’ contact events at the millisecond timescale in the early stages of virus–cell interactions.

### Rapid multiscale functional imaging in living organisms

Optically reading out the activity from many neurons in behaving animals is another example of multiscale imaging, often requiring rapid interrogation of large volumes with (sub)cellular resolution. Given the importance of functional imaging in neuroscience, there is a long history of optical microscopy development (Ji et al. 2008; Wu et al. 2013a; Winter and Shroff 2014) aimed at improving its depth penetration, speed, and spatial resolution. LSFM and multiphoton microscopy are particularly well suited to functional imaging, as we describe by considering two recent studies (Voleti et al. 2019; Demas et al. 2021).

**Fig. 4:**
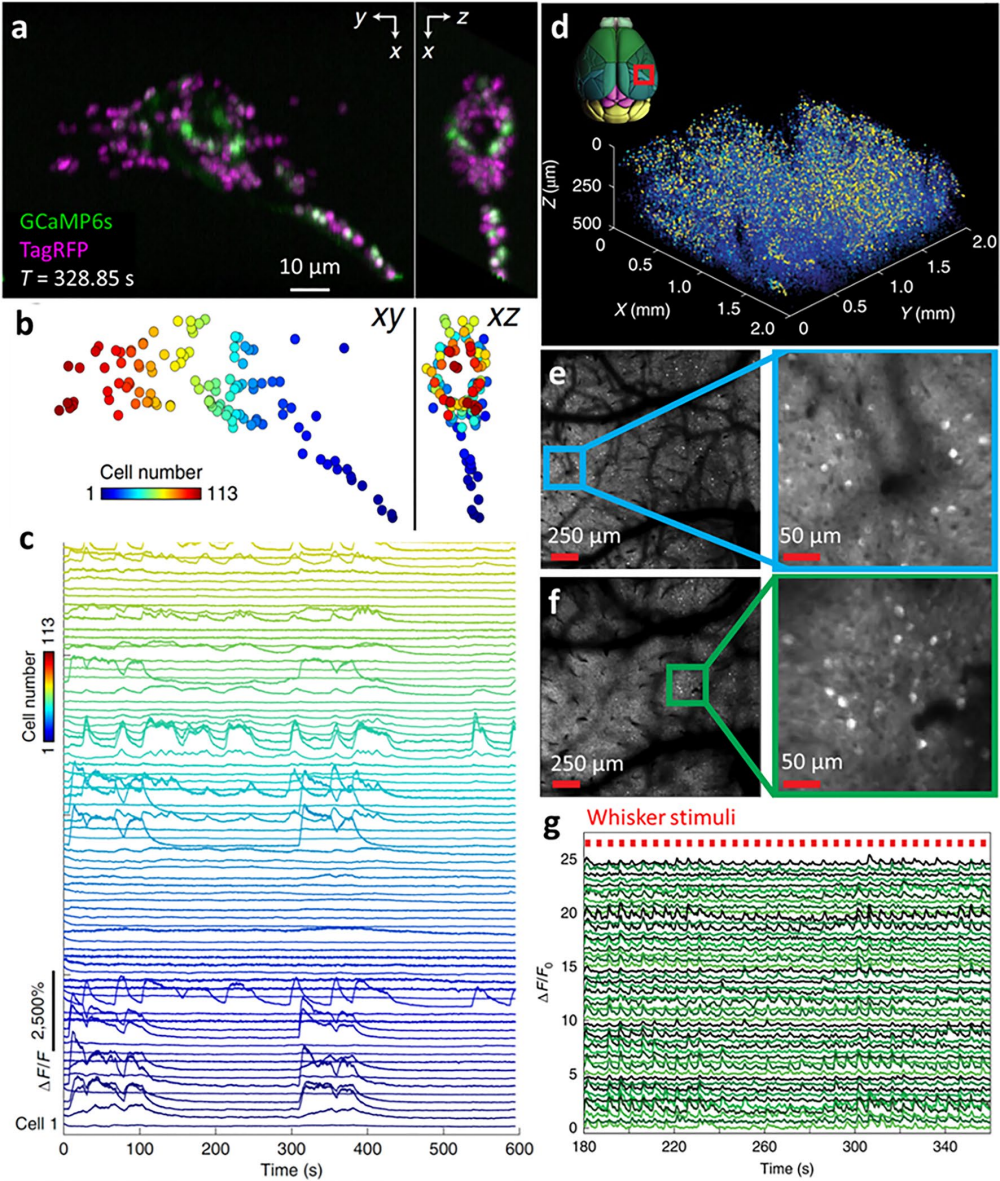
Rapid multi-scale functional imaging in living organisms. **a** Lateral (*left*) and axial (*right*) maximum intensity projections highlighting nerve ring region from a living, immobilized *C. elegans* young adult (NLS-GCaMP6s/TagRFP), acquired with SCAPE 2.0 at 5.96 volumes per second. **b** 113 neurons were identified and tracked in 3D space over 10 min and are ordered and color-encoded along the rostral-caudal axis. **c** Extracted raw GCaMP6s fluorescence time courses over 10 min. **d** Volumetric imaging (spanning ~ 2 × 2 × 0.5 mm^3^) of neural activity at 6.7 Hz in jGCaMP7f-expressing mice, acquired with light beads microscopy. 3D rendering of extracted neuron spatial coordinates and maximum projected activity for a 9-min recording. The* red rectangle* in the transverse brain image indicates the region over which responses from 70,275 neurons were recorded. **e**, **f** Mean projection images at 144- and 482-μm depths, respectively, with higher magnification views at right. **g** Representative time series of 50 whisker-tuned neurons. Occurrences of the whisker stimulus are denoted by* red marks*. **a**–**c** were reprinted with permission from ref (Voleti et al. 2019), and **d**–**g** reprinted with permission from ref (Demas et al. 2021)

SCAPE-2.0 (Voleti et al. 2019) is an improved implementation of swept, confocally aligned planar excitation (SCAPE) microscopy (Bouchard et al. 2015) equipped with a high-speed intensified camera. SCAPE is an LSFM implementation that uses a single, motionless objective lens to introduce an oblique sheet for illumination, which is scanned throughout the sample volume via a large aperture galvanometer mirror. The galvanometer mirror also de-scans the fluorescence such that the image of the oblique light sheet is stationary, thereby achieving volumetric imaging without requiring any sample or objective lens motion. For example, SCAPE-2.0 enabled a volumetric rate of 100 Hz when imaging 3-day post-fertilization zebrafish embryos expressing GFP and DsRed (volume spanning 640 × 148 × 127 voxels), or more than 300 volumes per second when performing dual-color cardiac imaging of the zebrafish heart over a smaller subvolume. Although the emission pathway of SCAPE 2.0 is complex, leading to lower resolution and greater light loss than other LSFM implementations, SCAPE 2.0’s speed and micron-scale resolution is well suited for many multiscale imaging applications. For example, SCAPE-2.0 facilitated functional imaging of the head of an immobilized young adult *C. elegans* worm expressing nuclear-localized GCaMP6s and TagRFP at 5.96 volumes per second for 10 min (Fig. 4a). This imaging speed, along with the comparatively low phototoxicity compared to spinning disk confocal microscopy, facilitated 4D tracking of the TagRFP-labeled nuclei and extraction of the associated GCaMP fluorescence signals from 113 neurons within the worm head (Fig. 4b). Neurons exhibited distinct patterns of calcium activity (Fig. 4c). Analyzing the correlations between neurons revealed that the nervous system appeared to switch between six distinct states during the recording.

Although SCAPE-2.0 enables rapid functional imaging over relatively large volumes, its penetration depth is limited to relatively transparent tissue. Multiphoton microscopy is better suited to applications in scattering tissue. Common multiphoton microscopes use high-repetition-rate lasers (e.g., 80 MHz), which often oversample cell bodies in the lateral plane and use multiple pulses per pixel to improve SNR, resulting in slow frame rates. To better sample the imaging volume while improving SNR and frame rate, a ‘light beads microscope (LBM)’ based on a low-repetition-rate laser (4.7 MHz) was implemented. LBM splits each femtosecond pulse into 30 copies using a cavity-based multiplexing approach, delaying each copy in time and focused it into a different depth, thus forming a column of ‘light beads’ (Demas et al. 2021). Illuminating with the 30 ‘beads’ enables near-simultaneous recording of activity over an imaging depth of 465 µm with ~ 16-μm axial separation between imaging planes. By effectively using the deadtime between laser pulses (~ 200 ns) to collect the signal from each axial plane, volumetric recording at 1.41 × 10^8^ voxels per second is possible. LBM was used to record calcium activity from a volume of ~ 2.0 × 2.0 × 0.5 mm^3^, containing a population of ~ 70,000 neurons, in the posterior parietal region of a jGCaMP7f-expressing mouse neocortex imaged at 3-μm lateral voxel sampling and ~ 6.5-Hz volumetric rate (Fig. 4d–g). With the ability to tune the lateral sampling, LBS is well suited to multiscale imaging of the mouse cortex at variable spatiotemporal resolution, enabling e.g., sub-cellular-resolution recordings within a ~ 0.6 × 0.6 × 0.5 mm^3^ volume at ~ 10 Hz and 1-μm lateral voxel sampling; cellular-resolution recordings within a ~ 3 × 5 × 0.5 mm^3^ volume containing more than 200,000 neurons at ~ 5 Hz, and recordings of populations of ~ 1 million neurons within a ~ 5.4 × 6 × 0.5 mm^3^ volume at ~ 2 Hz and 5-μm lateral voxel sampling.

### Adaptively correcting for changes in sample size and sample-induced aberrations improves imaging in tissue

Multiscale imaging is especially challenging when the sample itself changes in scale over the imaging experiment. For example, when imaging mouse embryos over 48 h from gastrulation to organogenesis, the sample expands more than 250-fold in volume, dramatically changing in shape (e.g., from ~ 200 µm to ~ 1.3 mm in linear dimension) and morphology. Such a dramatic change in sample size and shape confounds imaging with conventional microscopes, most obviously because the sample easily drifts out of the initial field of view. Imaging difficulties are further compounded by the time-varying optical properties of the sample. These problems were addressed in a recent study (McDole et al. 2018) that combined multiview light-sheet microscopy (Tomer et al. 2012; Chhetri et al. 2015) with an improved, automated method for adjusting the imaging parameters to optimize image quality [‘AutoPilot’ (Royer et al. 2016)], thereby adapting the microscope to the changing sample while maintaining viability over days of continuous imaging spanning gastrulation to early organogenesis. This system optimized spatial resolution by continuously computing and analyzing maximum-intensity projections of the imaging volume along multiple axes, monitoring image quality metrics within the context of a dynamic, geometric model of the sample, and adjusting the positions of objectives and tube lenses with motorized stages to compensate for specimen induced defocus, spherical aberration, and movement. Three-dimensional (3D) movement and growth of the embryo were monitored by tracking cells expressing ubiquitous nuclear reporter (mKate2-NLS) with a novel cell-tracking framework.

**Fig. 5:**
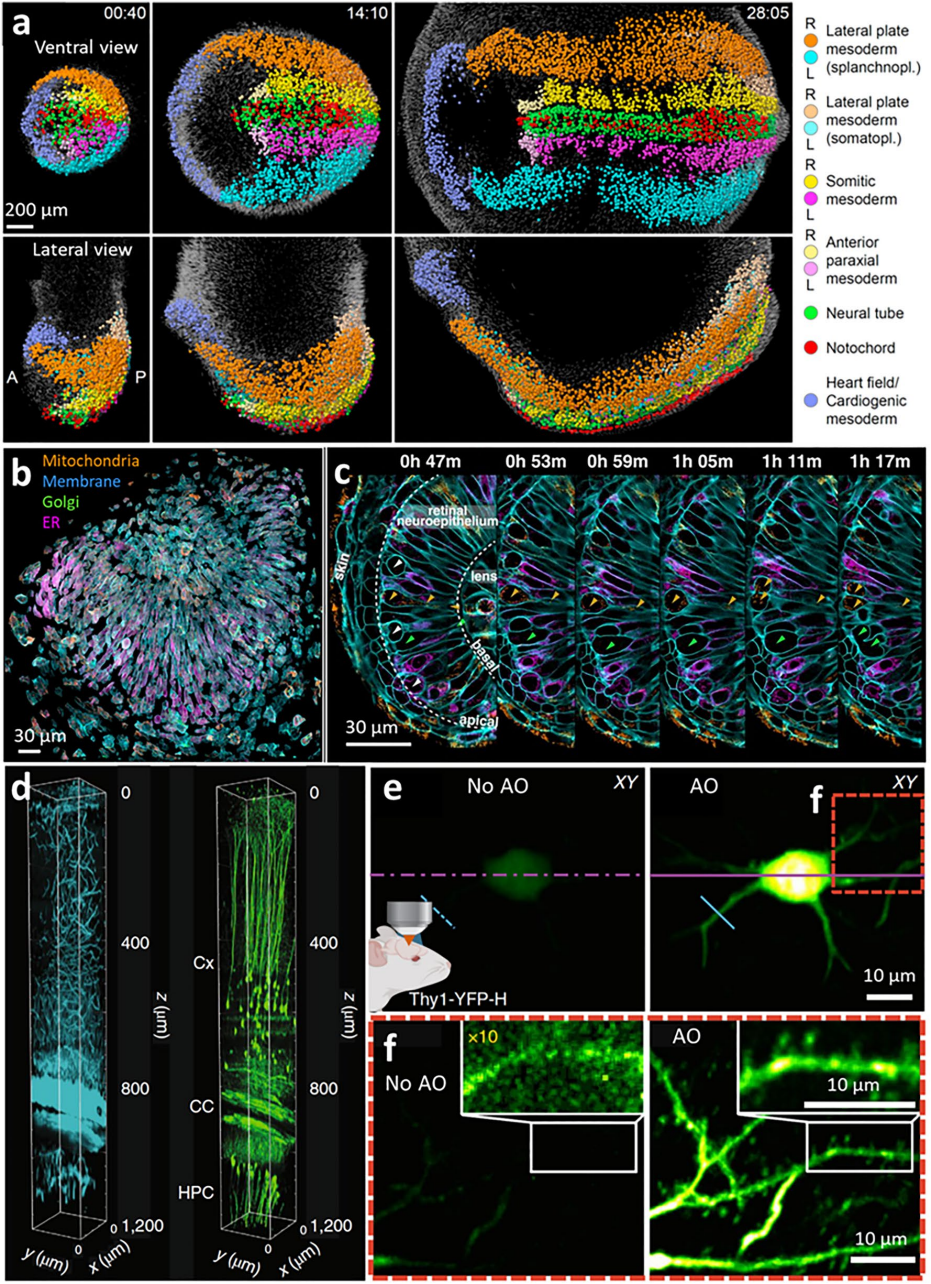
Adaptively correcting for sample size and aberrations when using light-sheet and three-photon microscopy improves imaging at large length scales. **a** Reconstruction of mouse embryo at single-cell resolution, acquired with adaptive light sheet microscopy. Three time points (hh:mm) selected from an experiment spanning mid/late-streak stage to early somite stage. Here, tracks are derived from combining the cell-tracking framework with a machine learning module (TGMM 2.0) and statistical vector flow (SVF) analysis. The dynamic fate map was created by labeling tissues in the image data at the last time point, transferring labels to SVF objects (*spheres*) and propagating labels backwards in time. **b**, **c** Tiled adaptive optics (AO) lattice light sheet microscopy permits detailed examination of cellular morphology and organelle distributions across the eye of a developing zebrafish embryo 24–27 h post fertilization. Here, computationally separated cells are shown spanning the data in (**b**), with organelles colored as indicated. Orthoslices at six different time points are shown in (**c**) highlighting cell divisions (*white* and* green arrowheads*,* left panel*) at the apical surface of the retinal neuro-epithelium and mitochondria (*orange arrowheads*) present from the apical to the basal surface in one dividing cell. **d** ECG-gated AO 3PM at 1300-nm excitation wavelength in EGFP–Thy1(M) mouse visual cortex and hippocampus, shown as 3D reconstruction of an image stack of third-harmonic signal (*cyan*) and GFP-labeled neurons (*green*). Aberration correction is performed via a modal-based indirect wavefront sensing approach. Intravital motion artifacts are reduced with a real-time ECG-gated image acquisition scheme that synchronizes scanners to the cardiac cycle of the mouse. **e** Maximum intensity projection of a neuron in the mouse cortex (Thy1-YFP-H), at 747–767 μm below dura, under 1300-nm three-photon excitation, without and with AO based on a zonal aberration measurement method. **f** Higher-magnification views of the red square in **e**, 751–767 μm below dura, without and with AO.* Insets* show higher magnification views of the dendrite shown in* white rectangles* in **f**. 10 × digital gain was applied to the No AO inset to increase visibility. Reprinted with permission from ref (McDole et al. 2018) for (**a**), ref (Liu et al. 2018) for (**b**, **c**), ref (Streich et al. 2021) for (**d**), and ref (Rodriguez et al. 2021) for (**e**, **f**)

This adaptive imaging framework was used to visualize mouse embryos at single-cell resolution spanning from the mid/late-streak stage to the early somite stage, enabling the creation of a dynamic fate map for all individual cells (Fig. 5a). Optimizing the spatial resolution by minimizing defocus proved essential for this goal, which was improved compared to previous systems (e.g., across the whole specimen, detection defocus errors were 2.99 ± 1.34 µm for non-adaptive light-sheet microscopy, 1.90 ± 0.81 µm with the previous AutoPilot framework, and only 0.06 ± 0.04 µm with the new platform).

As discussed above, AO provides another framework to compensate for sample-induced aberrations, and has been used effectively in conjunction with lattice light sheet microscopy (Chen et al. 2014) [AO-LLSM (Liu et al. 2018)] to obtain diffraction-limited resolution when imaging within live multicellular organisms. In this study, aberrations in both the excitation and emission paths were corrected with direct wavefront sensing, i.e., using a Shack–Hartmann sensor to measure the wavefront from a point-like light source (“guide star”) created by two-photon excitation. The nonlinear guide star was scanned to sample the average wavefront over the imaging field, thereby generating an average correction more accurate than single-point correction (Wang et al. 2014) and simultaneously reducing the photon load compared to dwelling at a single point. With a correction time down to 70 ms, this AO method is well matched to the speed and gentleness of LLSM. This advance permitted detailed examination of cellular morphology and organelle distributions across the eye of a developing zebrafish embryo 24–27 h post fertilization (Fig. 5b, c). Other applications of AO-LLSM included in vivo 3D imaging of clathrin-mediated endocytosis and spinal cord neural circuit development in zebrafish embryos.

Direct wavefront sensing techniques are only useful when sufficient ‘ballistic’ (unscattered) fluorescence reaches the sensor, thereby providing an accurate estimate of the wavefront. In conditions where the ballistic signal from the target of interest is weak, a second, brighter fluorophore may be introduced to boost the signal (Wang et al. 2015). Even this strategy fails in very thick, scattering samples. In these cases, the wavefront can still be estimated from a sequence of images with changes applied to the adaptive element (‘indirect wavefront sensing’). These sensorless AO systems include ‘modal’ methods, where the aberrations are estimated with using a series of modes defined over the whole pupil, and ‘zonal’ approaches, in which the pupil is separated into different zones that are modulated separately. Both approaches are very effective when applied to imaging deep, scattering brain tissues with three-photon microscopy.

For example, tissue scattering, optical aberrations, and motion artifacts were recently mitigated by combining three-photon excitation, modal-based sensorless AO, and active electrocardiogram (ECG) gating (Streich et al. 2021) in an in vivo mouse model. Image acquisition was paused during peaks in the ECG, reducing intraframe motion artifacts and resulting in better visualization of fine structures such as individual spines. This combination of techniques improved deep tissue imaging to the point that near-diffraction-limited imaging of neuronal structure (EGFP–Thy1(M) label) and third-harmonic generation contrast of an in vivo mouse brain was achieved along an entire cortical column ~ 1.2–1.4 mm in depth (the edge of the mouse CA1 hippocampus, Fig. 5d, e). A related study combining three photon excitation with zonal-based sensorless AO (Rodriguez et al. 2021) enabled the in vivo study of fine neuronal processes and synapses in deep cortical layers ~ 750 μm below the dura of the mouse brain (Fig. 5f, g). Other multiscale imaging demonstrations with this three-photon AO system included functional imaging of calcium activity in jGCaMP7s-expressing neurons of the dorsal horn ~ 300 μm below the dura in the mouse spinal cord, in response to cooling stimuli applied to the hindlimb skin.

## Improving multiscale imaging with computation

### Image restoration with deep learning

**Fig. 6:**
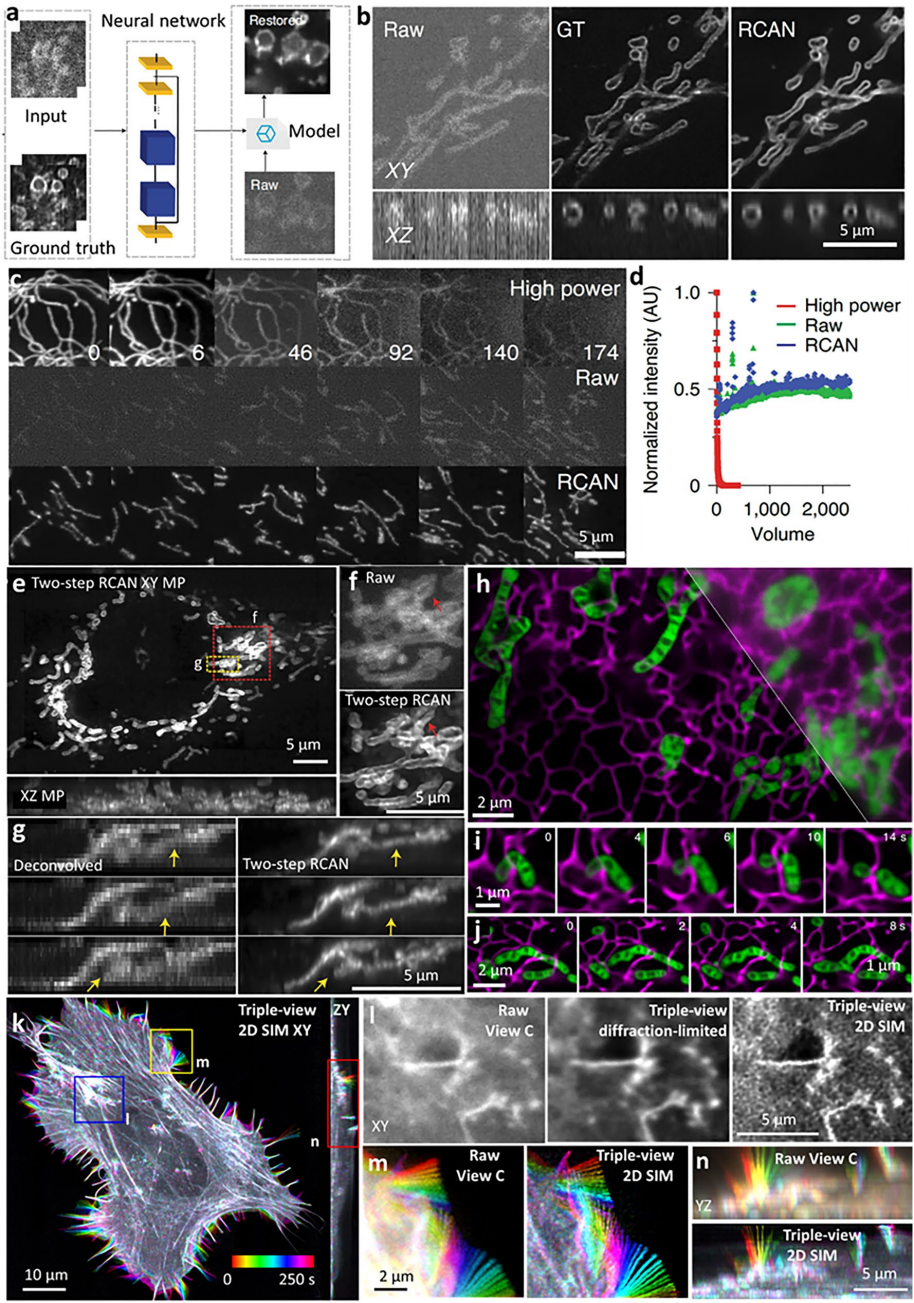
Image restoration with deep learning. **a** With suitable training data, a neural network may be used to denoise images. **b** Lateral (*upper*) and axial (*lower*) images of a fixed U2OS cells expressing mEmerald-Tomm20 imaged via iSIM, comparing noisy raw iSIM data acquired with low-intensity illumination (*left*), deconvolved GT data acquired with high-intensity illumination (*middle*), and RCAN output (*right*) given raw input. **c** RCAN denoising enables the collection of thousands of iSIM volumes without photobleaching. Mitochondria in live U2OS cells were labeled with pShooter pEF-Myc-mito-GFP and imaged with high- (360 W cm^–2^) and low- (4.2 W cm^–2^) intensity illumination.* Top row*: selected examples at high illumination power, illustrating severe photobleaching.* Middle row*: selected examples from a different cell imaged at low illumination power, illustrating low SNR (Raw).* Bottom row*: RCAN output given low SNR input.* Numbers in top row* indicate volume #. **d** The graph quantifies the normalized signal in each case in (**c**); ‘jumps’ in Raw and RCAN signal correspond to manual refocusing during acquisition. **e**–**g** Two-step RCAN process (RCAN denoising followed by RCAN expansion) is applied to deconvolved iSIM images to generate expansion predictions. Images from live U2OS cells expressing EGFP-Tomm20 were acquired with iSIM, deconvolved and input into the two-step RCAN process. **e** Overview of lateral and axial maximum intensity projections of first volume in time series from two-step RCAN prediction. **f** Higher-magnification views of red rectangular region in (**e**), comparing raw iSIM and RCAN prediction.* Red arrows* highlight mitochondria better resolved with RCAN than iSIM. **g** Higher-magnification views of axial slice corresponding to yellow rectangular region in (**e**), comparing deconvolved iSIM input (*left*) and two-step RCAN output (*right*).* Yellow arrows* highlight mitochondria that are better resolved with RCAN output than input data. **h**–**j** Super-resolution images of ER (*magenta*) and mitochondrial cristae (*green*) in live U2OS cells, acquired with GI-SIM and generated by DFCAN-SIM (ER) and DFGAN-SIM (mitochondria), respectively.* Top right*: a fraction of the corresponding widefield image averaged from raw SIM images. Time-lapse images showing mitochondrial fission (**i**) and fusion (**j**) events occurring at ER–mitochondria contact sites. **k**–**n** Deep learning and joint deconvolution produces 2D super-resolution images from diffraction-limited input. Example 2D SIM maximum intensity projection of U2OS cell expressing Lifeact tdTomato, volumes every 10 s, over 100 time points. Images are color-coded to indicate temporal evolution. **l**–**n** Comparative higher-magnification view of blue, yellow, red regions in (**k**), color-coded in (**m**, **n**) to illustrate the filopodial dynamics. Reprinted with permission from ref (Chen et al. 2021) for (**a**–**g**), ref (Qiao et al. 2021) for (**h**–**j**), and ref (Wu et al. 2021) for (**k**–**n**)

Deep learning (LeCun et al. 2015) has emerged as a powerful method for image restoration, allowing a ‘softening’ of the imaging tradeoffs discussed above. With suitable training data, neural networks may be trained and subsequently applied for denoising (Weigert et al. 2018; Chen et al. 2021) (Fig. 6a), deconvolution (Guo et al. 2020; Li et al. 2022), or resolution enhancement (Ouyang et al. 2018; Wang et al. 2019; Qiao et al. 2021; Chen et al. 2021). Multiple recent examples illustrate the potential of these methods for multiscale imaging.

Three-dimensional residual channel attention networks (3D RCAN (Chen et al. 2021)) denoise and sharpen fluorescence microscopy volumes, quantitatively outperforming previous network architectures (Weigert et al. 2018; Ledig et al. 2017; Wang et al. 2018b) when denoising low SNR images of labeled mitochondria (Fig. 6b), actin, endoplasmic reticulum, Golgi, lysosomes, and microtubules acquired with instant SIM (York et al. 2013) (iSIM). ISIM is a rapid super-resolution technique capable of enhancing resolution ~ twofold over the diffraction limit without sacrificing temporal resolution, possessing the additional advantage that this resolution gain may be achieved in transparent tissue ~ 10 × thicker than a single cell (York et al. 2012). A significant drawback of iSIM is that it illuminates the sample volumetrically, leading to photobleaching and photodamage that ultimately limit the temporal scale (i.e., duration) of the experiment. One way around this problem is to lower the illumination intensity to the point that photobleaching is negligible; unfortunately, the resulting SNR is usually so low that the data become unusable.

Applying a denoising 3D RCAN on noisy iSIM images of U2OS cells labeled with a mitochondrial marker addressed the SNR challenge, enabling super-resolution imaging over thousands of image volumes (tens of thousands of images; image volumes acquired every 5.6 s over 4 h, Fig. 6c), without detectable photobleaching and even an apparent increase in fluorescence signal over the course of the recording (Fig. 6d). The restored image quality was sufficiently high that individual mitochondria could be manually segmented. In a second example, applying a denoising 3D RCAN on low SNR images of U2OS cells labeled with lysosomal and mitochondrial markers improved SNR to the extent that mitochondrial fission and fusion events near lysosomal contacts could be quantified, which was otherwise impossible on the raw input data. Both examples highlight the capacity to effectively extend SNR and temporal scale beyond the capabilities of the base microscope.

A second application enabled by deep learning is the improvement of spatial resolution, permitting the use of lower illumination dose microscopes, often advantageous when imaging living samples. For example, given comparatively higher-resolution STED microscopy ground truth, a 3D RCAN was used to improve the lateral resolution of noisy confocal microscopy images by ~ 2.5-fold. This capability was useful in improving the resolution of low SNR, volumetric time-lapse imaging of cell division (Chen et al. 2021), which is challenging to observe using higher-resolution STED microscopy. In another example, expansion microscopy (Chen et al. 2015) (a method that physically enlarges dead specimens, enabling improved resolution) data was used as ground truth to train a 3D RCAN to improve the volumetric resolution of iSIM. By combining this network with another denoising 3D RCAN and applying the two-step deep learning scheme to volumetric time-lapse sequences of living U2OS cells labeled with EGFP-Tomm20 (Fig. 6e–g), closely packed mitochondria that were badly blurred in the axial direction could be discerned.

In a parallel study, RCANs were augmented by leveraging the frequency content differences across image features to better predict the high-frequency information present in super-resolution images, resulting in ‘deep Fourier channel attention networks’ [DFCAN (Qiao et al. 2021)]. The DFCAN enabled high-quality TIRF-SIM (Kner et al. 2009), grazing-incidence SIM [GI-SIM (Guo et al. 2018)], and nonlinear SIM (Li et al. 2015) reconstructions from low SNR input. In some cases, the DFCAN enabled tenfold longer multicolor live cell super-resolution recordings than would have been possible without denoising. The DFCAN predictions provided detailed views of mitochondria undergoing fission/fusion in the vicinity of ER–mitochondria contact sites (Fig. 6h–j).

The spatiotemporal scales accessible to a recently developed multiview confocal super-resolution microscope were also enhanced using deep learning (Wu et al. 2021). In this case, collection of 15 diffraction-limited image volumes can provide 3D resolution enhancement using SIM reconstruction algorithms. This relatively large amount of raw data impedes super-resolution imaging over extended duration due to the extra illumination imparted to the sample; it also compromises temporal resolution relative to the base confocal multiview microscope. To address these challenges, an RCAN model was trained to predict 1D super-resolution images from diffraction-limited input. Then, by digitally rotating an input volume and reapplying the trained model, 1D resolution could be improved along any lateral direction lying within the focal plane. By combining the six digitally rotated, 1D super-resolved views derived from a single confocal input image, a deep learning prediction with isotropic 2D resolution enhancement could be obtained. Applying this method to three views acquired with the base multiview confocal microscope improved 3D SIM imaging over multiple scales, as demonstrated by visualization of actin dynamics in a U2OS cell over 100 volumes (Fig. 6k–n), and the nervous system in L2-L4 stage *C. elegans* larvae.

### Multiscale imaging of fine dynamic processes with event-triggered acquisition

Another strategy that is well suited for multiscale imaging, improving imaging throughput and quality for a region of interest (ROI), is ‘event-driven acquisition (EDA) (Alvelid et al. 2021; Mahecic et al. 2021). Unlike the adaptive strategies above that attempt to globally improve image quality, EDA instead attempts to automatically switch between imaging modalities by monitoring real-time changes in the sample (e.g., intensity spikes, local movement, and morphological changes), zooming into the ROI only when necessary.

**Fig. 7:**
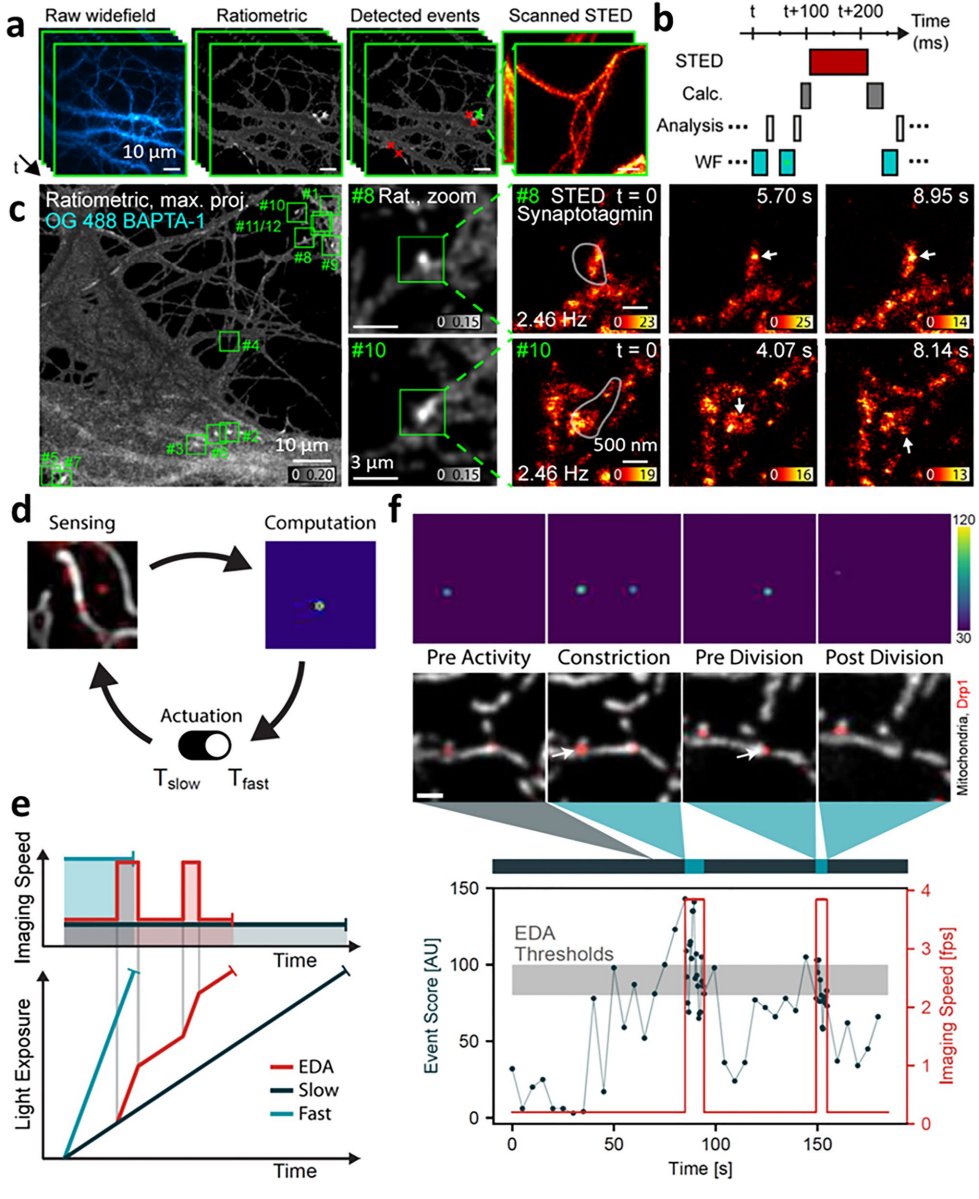
Image-based event triggering improves multiscale imaging of fine dynamic processes. **a** Schematic for event triggered STED (etSTED): widefield calcium imaging of Oregon Green 488 BAPTA-1 in neurons, corresponding ratiometric images, detected events, and local etSTED images of SiR-tubulin. The* green cross* indicates the ratiometrically brightest detected event that triggered STED imaging and the* red crosses* indicate additionally detected events in the same widefield frame. **b** Timeline of typical etSTED experiment, with widefield, analysis pipeline, overhead, and STED imaging time indicated. **c** etSTED experiments, imaging protein synaptotagmin-1(syt-1) conjugated to Abberior STAR635P. Syt-1 is the calcium sensor that triggers vesicle release. Maximum-projected ratiometric image of 12 detected events (calcium spike events) from an experiment spanning 4 min 24 s (*left*,* green squares* show location of detected events), zoomed-in views of the ratiometric image in two detected events (*center*), and 2.46-Hz etSTED timelapse of syt-1 in a 3 × 3 μm^2^ field of view, showing dynamic activity of the synaptic vesicles during calcium sensing. **d** Schematic of event-driven acquisition (EDA) framework, combining real-time, neural network-based recognition of events of interest (e.g., mitochondrial division in the presence of dynamin related protein—Drp1) with automated control of the imaging parameters in imaging (e.g., frame rate). The EDA feedback control loop between the sample and the acquisition parameters is composed of three main parts: (1) sensing by image capture to gather information from the sample, (2) computation to detect events of interest to generate a probability map, and (3) adaptation of the acquisition parameters in response to the sample. **e** Schematic representation of the trade-off between the imaging speed and light exposure over the duration of an imaging experiment. The total amount of photon budget available (*shaded areas*) stays the same for all techniques. **f*** Top*: Examples of frames capturing events of interest (mito-Emerald in* grey*, Drp1-mCherry in* red*) that triggered a change in imaging speed and the corresponding probability maps.* Bottom*: The corresponding event probability (computation,* black*) as a function of time obtained during an EDA-guided iSIM imaging experiment, and the adaptive imaging speed (actuation,* red*). This self-driving microscope captures the mitochondrial divisions at imaging rates that match their dynamic time scale, while preserving the sample from unnecessary illumination and extending the accessible imaging duration. **a**–**c** were reprinted with permission from ref (Alvelid et al. 2021), and **d**–**f** reprinted with permission from ref (Mahecic et al. 2021)

In a first example, event-triggered STED microscopy (etSTED) (Alvelid et al. 2021), rat hippocampal neurons containing fluorescently labeled proteins were imaged with high spatiotemporal resolution (~ 30 nm, < 40 ms per frame, 2.5 Hz) in small ROIs exhibiting calcium activity (Fig. 7a, b). Rapid widefield imaging enabled the detection and localization of calcium intensity spikes at the millisecond timescale. The location of ROIs of interest were fed into a rapid analysis pipeline to trigger correlated STED microscopy acquisition within ~ 20 ms of the detected event, thereby facilitating correlative super-resolution imaging of actin, tubulin, or synaptotagmin-1 within synaptic vesicles (Fig. 7c) in the ROI. Rearrangements of the shape, density, and connectivity of densely packed synaptic vesicles, including individual vesicle dynamics and disassembly of the clusters were captured with et-STED. Finding such rare, rapid events with conventional STED microscopy (i.e., imaging the entire field of view) would be difficult due to its relatively slow imaging speed and phototoxicity.

In a second example of EDA (Mahecic et al. 2021), neural-network based recognition of events of interest was used to automatically control the frame rate of iSIM (Fig. 7d, e), adjusting it as necessary to capture mitochondrial constriction and division in Cos7 cells (Fig. 7f). Although the Dynamin-related protein Drp1 promotes mitochondrial fission, its presence alone is not predictive of future divisions. Thus, simple thresholding of Drp1 signal alone is insufficient to predict constriction. Instead, a neural network was trained on 29,600 images of Cos-7 cells expressing mitochondrion-targeted TagRFP and Drp1-Emerald, and frames marking the respective division locations (ground truth) to generate a probability map predicting the most pronounced constriction site. This map proved more accurate than simple thresholding and was thus incorporated into an automated pipeline that increased iSIM imaging speed to better sample the process of division. Since temporal sampling was adjusted only when needed, EDA-guided acquisition made better use of the finite photon budget than conventional iSIM imaging, lessening photobleaching and photodamage. In another application, EDA-guided iSIM better captured bacterial cell division events than conventional iSIM imaging.

### Creation of multiscale atlases by combining information from multiple samples

Although live imaging methods continue to advance, seeing everything of interest across all spatiotemporal scales in a single experiment remains difficult, if not impossible. For model organisms with stereotypical behavior, anatomy, or development, it is sometimes possible to pool multiple datasets from distinct experiments to create a composite, statistical model spanning multiple scales in space or time. Such ‘atlases’ are particularly powerful in developmental biology, as they may be used to resolve development in ‘4D’, observe how biological dynamics differ across individual organisms and how these differences compare to the mean, and facilitate comparisons between wild-type and mutant phenotypes. We present two examples of multiscale atlases that involve very different imaging challenges.

**Fig. 8:**
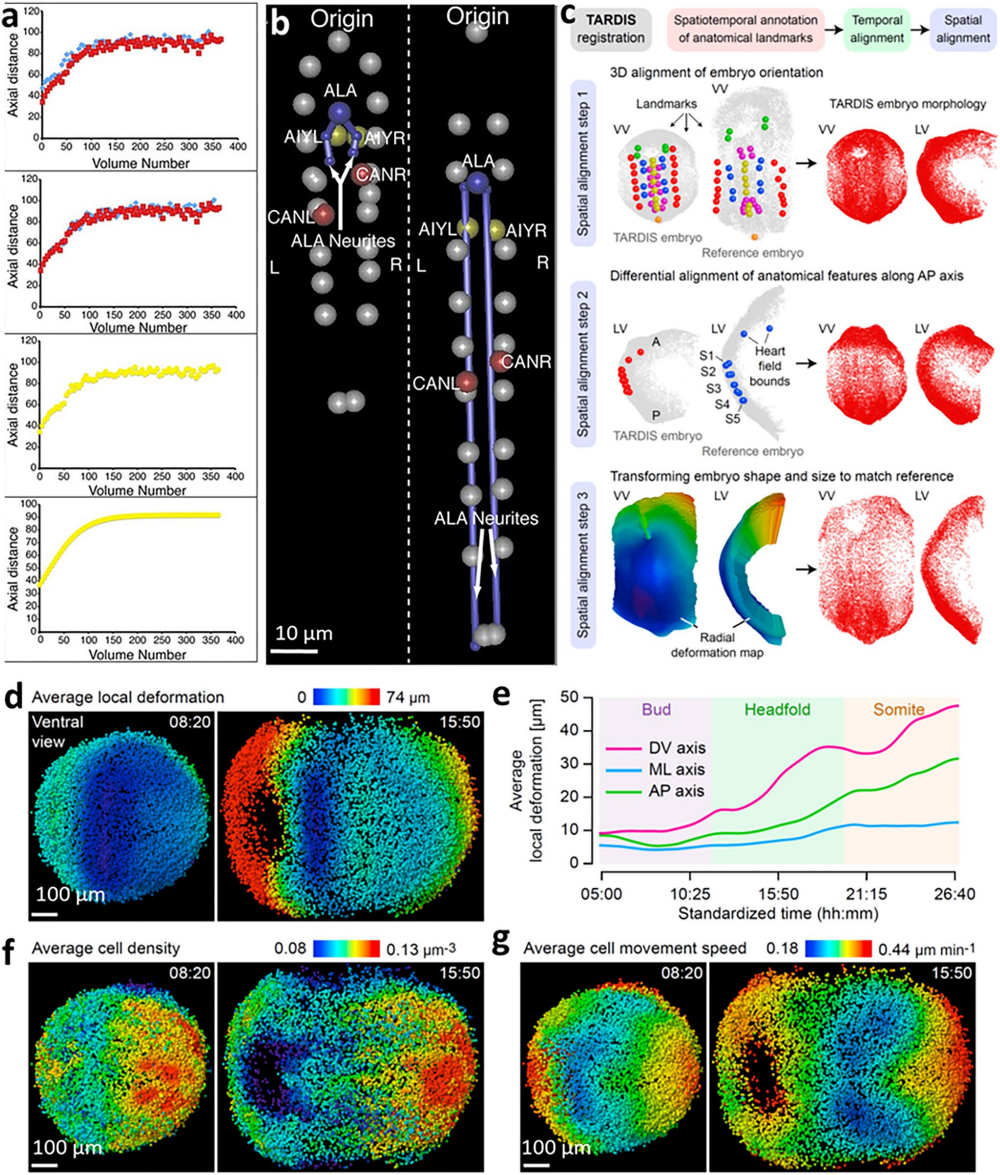
Combining information from multiple samples allows the creation of dynamic, multiscale atlases. **a** Alignment of data from different *C. elegans* embryos.* Top to bottom*: (1) axial seam cell nuclear trajectories from different embryos are similar in shape, but shifted in time; (2) shifting in time aligns the trajectories; (3) averaging the shifted trajectories; (4) fitting the shifted trajectories. Examples show the shifting, averaging, and fitting process for two embryos. **b** Composite model of seam cell nuclear movement and neuronal development in the *C. elegans* embryo, established from four embryos. Shown are two typical time points at early (*left*, about 8-h post fertilization) and late (*right*, about 13 h post fertilization) stage in the elongating embryo. Canal associated neurons (CANL, CANR) moved faster than adjacent seam cell nuclei, suggesting a more 'active' mode of migration. Seam cell nuclei:* gray spheres*; ALA cell body:* blue sphere*; ALA neurites:* blue lines*; AIY cell bodies:* yellow spheres*; CAN cell bodies:* red spheres*. **c**–**g** Stereotypy of local cell dynamics across four mouse embryos aligned with spatiotemporal registration TARDIS (time and relative dimension in space) method. **c** Overview of TARIDS: embryos are aligned in time using manual annotations, then aligned in space by rigid registration to a reference embryo using spatial landmarks (step 1), differential alignment of anatomical features along anterior–posterior axis (step 2), and transformation of their shape and size to match the reference embryo (step 3).* Left*: examples of landmarks and transformation maps are shown.* Right*: resulting embryo morphology. Visualization (**d**) and quantification (**e**) of differences in local embryo shape across four rigidly aligned embryos. *DV* dorsoventral, *ML* mediolateral, *AP* anteroposterior. Average local cell densities (**f**) and average local cell movement speeds (**g**) are shown at two time points in the average embryo. **a**–**b** were reprinted with permission from ref (Christensen et al. 2015), and **c**–**g** reprinted with permission from ref (McDole et al. 2018)

*C. elegans* possesses a relatively simple nervous system (White et al. 1986) and invariant embryonic cell lineage (Sulston et al. 1983). Repeated cell divisions transform a single embryonic cell into 558 cells (222 neurons) in the hatched larva. Given the relatively small embryo size (50 × 40 × 30 μm^3^), it is possible to continuously follow cellular growth, division, and morphological change throughout the entirety of the 14-h embryogenesis period using light-sheet microscopy (Wu et al. 2011, 2013b). Although all neurites in the densely packed nervous system cannot be simultaneously resolved (due to the diffraction limit), it is possible to target GFP to sparse subsets of optically resolvable neurons, segment and track them, and incorporate the results from many such experiments on distinct embryos into a global model showing a systems-level, 4D view of brain development in the first half of embryogenesis (Santella et al. 2015; Moyle et al. 2021). In the latter half of embryogenesis, rapid movements (‘twitching’) and the embryo’s entangled posture confound atlas building. To tackle these difficulties, ‘untwisting’ software was developed to straighten the embryo and facilitate tracking of seam cell nuclei, neuronal cell bodies, and neurites in embryos (Christensen et al. 2015). After untwisting and temporal alignment (Fig. 8a), data from four *C. elegans* embryos were pooled to create a composite model showing movement of 20 seam cells, five neurons (AIYL/R, CANL/R, and ALA), and ALA neurites in an ‘average’ worm embryo with 2.5-min temporal resolution (Fig. 8b). The model revealed that (1) ALA and AIYL/R moved similarly to nearby seam cell nuclei; (2) CANL/R moved faster than adjacent seam cell nuclei, suggesting a more ‘active’ mode of migration; (3) the motion of ALA and CANL/R were considerably more variable between datasets than the seam cell nuclei; (4) ALA neurite growth outgrowth continued to occur for ~ 240 min after the other cells arrived their final positions at the end of elongation. While preliminary, the anatomical atlas is already yielding insights into behavior (Ardiel et al. 2021) and activity (Ardiel et al. 2017), and could be extended to encompass all neurons in the embryo.

More recently, a dynamic atlas of mouse development was assembled from four single embryos, yielding an extended duration model with 5-min temporal resolution and single-cell spatial resolution (McDole et al. 2018) over length scales spanning hundreds of microns to more than 1-mm. To jointly analyze data from multiple embryos, a spatiotemporal registration method—TARDIS (time and relative dimension in space, Fig. 8c)—was developed by combining manually annotated spatiotemporal landmarks and information derived from tracking cells with a machine learning based tracking framework. Using TARDIS, data from four embryos were registered in space and time, with a relatively small average registration error of 41.5 µm considering the large variations in shape and size between embryos. The average TARDIS embryo preserved the motion and morphology of different tissues as evident in individual embryos. For example, dramatic changes in tissue movement were observed during later stages when the embryo elongates axially, forming the anterior intestinal portal. From the average embryo, the average local deformation, cell density, and cell movement speed across development (Fig. 8d–g) could be quantified. Analyzing these metrics revealed that tissues in the anterior half of the embryo are driven primarily towards the anterior pole or inward along with the invaginating foregut pocket, that the highest degree of local shape variability between embryos was in the anterior-most region of the embryo, and that heart development displayed greater variability than almost any other region in the embryo.

## Summary: helpful themes in multiscale imaging

**Fig. 9:**
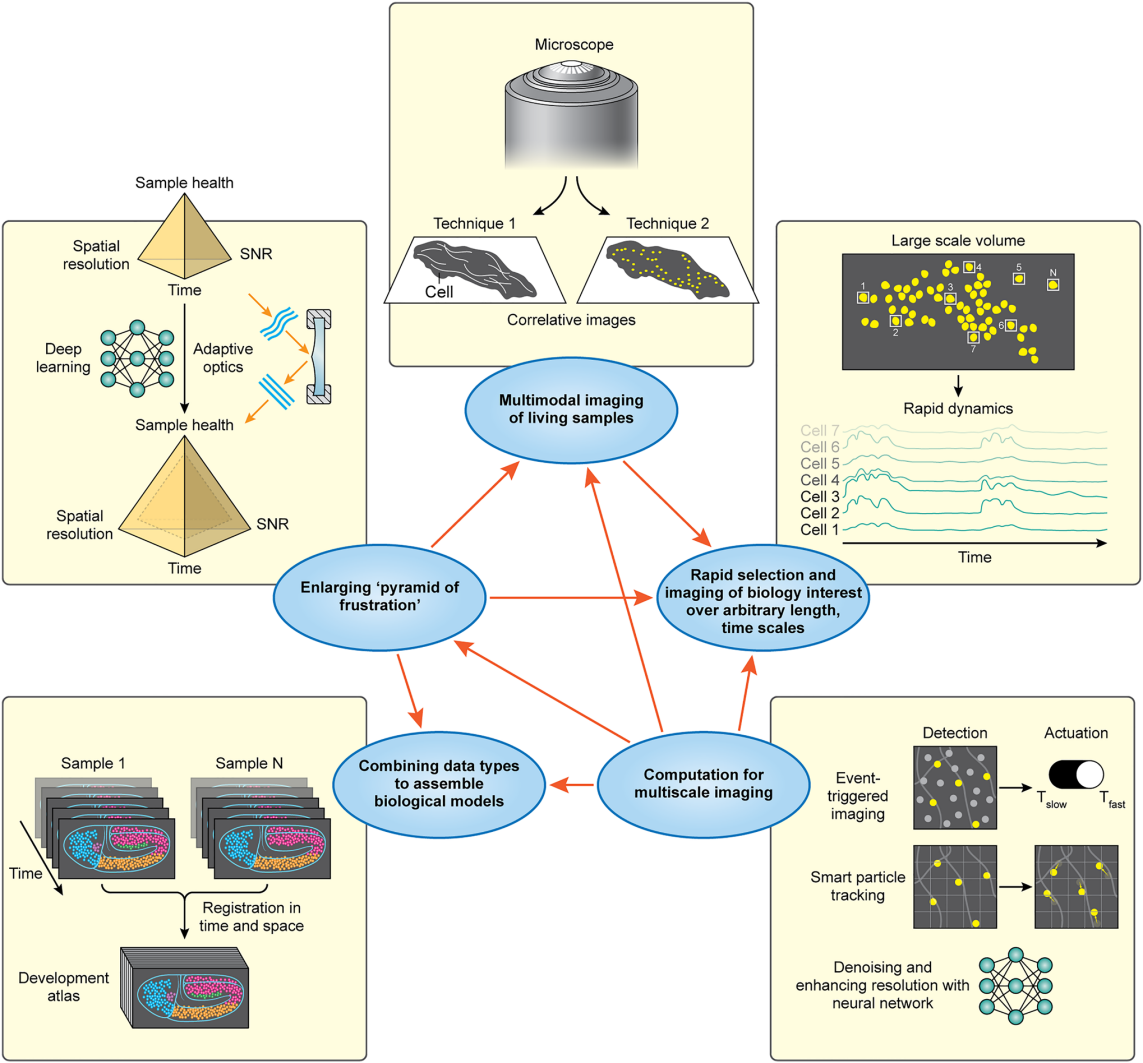
Key concepts for multiscale fluorescence imaging. Themes highlighted throughout this article, and inter-relationships between themes (*orange arrows*)

While diverse in application and methodology, the recent imaging efforts we surveyed (Figs. 3, 4, 5, 6, 7, 8) also reveal more general concepts that may be helpful in the quest to achieve multiscale imaging of living samples (Fig. 9). A key challenge in any approach is the ‘pyramid of frustration’: simultaneously optimizing spatial resolution, temporal resolution, phototoxicity, and SNR even for a single spatial or temporal scale is difficult. Recognizing this challenge motivates an obvious workaround: combine multiple modalities well suited to each spatiotemporal scale. This approach is particularly well suited for super-resolution imaging (Fig. 3a, b), which may 'miss the forest for the trees’ due to tradeoffs intrinsic to this class of methods. Diffraction-limited imaging methods also benefit from multimodality imaging, particularly in applications where a small region of interest must be imaged at a much faster rate than the surrounding larger context (Fig. 3c–f). Another class of applications that benefits from multimodal imaging is those in which the user must ‘find a needle in a haystack’, i.e., surveying a large field of view with modality #1 and then triggering higher-resolution acquisition with modality #2 (Fig. 7a–c).

In many cases, careful optimization of the microscope’s capabilities to best address the biological problem at hand allows the pyramid to be bypassed. The application of LBM and SCAPE to functional imaging (Fig. 4) exemplifies this approach, whereby the microscope technology is tailored to provide sufficient spatiotemporal resolution to rapidly target and image the biology of interest. Since the goal is to record activity traces at the single-cell level over many cells distributed in 3D, speed and field of view are prioritized over spatial resolution. In other cases, the parameter space within the ‘pyramid of frustration’ can itself be ‘enlarged’, ameliorating tradeoffs, and enabling more from the limited photon budget. Adaptively correcting for changes in sample size or time-/spatially varying changes in the sample’s optical properties (Fig. 5) provides one path to this goal. These methods require additional hardware and control algorithms to provide the requisite feedback. Alternatively, image restoration methods can circumvent underlying tradeoffs via computational means (Fig. 6), effectively predicting higher resolution or SNR from low-resolution, noisy data. We stress that the key word here is prediction. Particularly for newer, data-driven deep learning models, the practitioner is advised to consider the limitations imposed by the training data, as well as to perform controls to validate the predictions as necessary.

It is also worth remembering that computation can be broadly applied in service of multiscale imaging. Event-triggered acquisition (Fig. 7), multimodal imaging (Fig. 3c–f), and adaptive imaging strategies (Fig. 5) all are examples of ‘smart microscopy’(Scherf and Huisken 2015) that embed software algorithms to improve data acquisition. On the data analysis side, pooling multiple datasets allows the synthesis of data from different spatiotemporal scales (Fig. 8), enabling insights that may be difficult or impossible to glean from any single experiment.

Although this review has focused on optical and computational means to facilitate live multiscale imaging, we stress that many experiments are also (and perhaps more) limited by sample preparation. Continued development of bright and photostable optical probes and specific labeling strategies is key (Grimm and Lavis 2022), particularly to capture rare biological events. Improved sample mounting strategies, particularly for sustained imaging (Hof et al. 2021) in environments that mimic the 3D physiological environment (Keller et al. 2006) are also necessary. Finally, we believe that new mounting methods (Jakob et al. 2016) that facilitate cross-modality imaging will be important in exploring the ‘mesoscopic’ expanse that lies between single-cell and whole-animal scales.

## References

[CR1] Alvelid J, Damenti M, Testa I (2021). Event-triggered STED imaging. Nat Meth.

[CR2] Ardiel EL, Kumar A, Marbach J, Christensen R, Gupta R, Duncan W (2017). Visualizing calcium flux in freely moving nematode embryos. Biophys J.

[CR3] Ardiel EL, Lauziere A, Xu S, Harvey BJ, Christensen R, Nurrish S (2021). Stereotyped behavioral maturation and rhythmic quiescence in C.elegans embryos. eLife.

[CR4] Balzarotti F, Eilers Y, Gwosch KC, Gynna AH, Westphal V, Stefani FD (2017). Nanometer resolution imaging and tracking of fluorescent molecules with minimal photon fluxes. Science.

[CR5] Berning S, Willig KI, Steffens H, Dibaj P, Hell SW (2012). Nanoscopy in a living mouse brain. Science.

[CR6] Betzig E, Patterson GH, Sougrat R, Lindwasser OW, Olenych S, Bonifacino JS (2006). Imaging intracellular fluorescent proteins at nanometer resolution. Science.

[CR7] Bouchard MB, Voleti V, Mendes CS, Lacefield C, Grueber WB, Mann RS (2015). Swept confocally-aligned planar excitation (SCAPE) microscopy for high speed volumetric imaging of behaving organisms. Nat Photonics.

[CR8] Bratton BP, Shaevitz JW (2015). Simple experimental methods for determining the apparent focal shift in a microscope system. PLoS ONE.

[CR9] Chen BC, Legant WR, Wang K, Shao L, Milkie DE, Davidson MW (2014). Lattice light-sheet microscopy: imaging molecules to embryos at high spatiotemporal resolution. Science.

[CR10] Chen F, Tillberg P, Boyden ES (2015). Expansion microscopy. Science.

[CR11] Chen J, Sasaki H, Lai H, Su Y, Liu J, Wu Y (2021). Three-dimensional residual channel attention networks denoise and sharpen fluorescence microscopy image volumes. Nat Methods.

[CR12] Chhetri RK, Amat F, Wan Y, Hockendorf B, Lemon WC, Keller P (2015). Whole-animal functional and developmental imaging with isotropic spatial resolution. Nat Methods.

[CR13] Christensen R, Bokinsky A, Santella A, Wu Y, Marquina-Solis J, Guo M (2015). Untwisting the* Caenorhabditis elegans* embryo. Elife.

[CR14] Conchello J-A, Lichtman JW (2005). Optical sectioning microscopy. Nat Meth.

[CR15] De Luca GM, Breedijk RM, Brandt RA, Zeelenberg CH, de Jong BE, Timmermans W (2013). Re-scan confocal microscopy: scanning twice for better resolution. Biomed Opt Express.

[CR16] Demas J, Manley J, Tejera F, Barber K, Kim H, Traub FM (2021). High-speed, cortex-wide volumetric recording of neuroactivity at cellular resolution using light beads microscopy. Nat Methods.

[CR17] Denk W, Strickler JH, Webb WW (1990). Two-photon laser scanning fluorescence microscopy. Science.

[CR18] Ding Y, Vanselow DJ, Yakovlev MA, Katz SR, Lin AY, Clark DP et al (2019) Computational 3D histological phenotyping of whole zebrafish by X-ray histotomography. eLife 8:e44898 10.7554/eLife.44898 PMC6559789 31063133

[CR19] Fish KN (2009). Total internal reflection fluorescence (TIRF) microscopy. Curr Protoc Cytom.

[CR20] Foxley S, Sampathkumar V, De Andrade V, Trinkle S, Sorokina A, Norwood K (2021). Multi-modal imaging of a single mouse brain over five orders of magnitude of resolution. Neuroimage.

[CR21] Giannone G, Hosy E, Levet F, Constals A, Schulze K, Sobolevsky AI (2010). Dynamic superresolution imaging of endogenous proteins on living cells at ultra-high density. Biophys J.

[CR22] Grimm JB, Lavis LD (2022). Caveat fluorophore: an insiders' guide to small-molecule fluorescent labels. Nat Methods.

[CR23] Guo M, Liu Y, Su Y, Lambert T, Nogare DD, Moyle MW (2020). Rapid image deconvolution and multiview fusion for optical microscopy. Nat Biotechnol.

[CR24] Guo Y, Li D, Zhang S, Yang Y, Liu J-J, Wang X (2018). Visualizing intracellular organelle and cytoskeletal interactions at nanoscale resolution on millisecond timescales. Cell.

[CR25] Hell SW, Reiner G, Cremer C, Stelzer EHK (1993). Aberrations in confocal fluorescence microscopy induced by mismatches in refractive index. J Microsc.

[CR26] Hess ST, Girirajan TPK, Mason MD (2006). Ultra-high resolution imaging by fluorescence photoactivation localization microscopy. Biophys J.

[CR27] Hobson CM, Aaron JS (2022) Combining multiple fluorescence imaging techniques in biology: when one microscope is not enough. Mol Biol Cell (**in press**) 10.1091/mbc.E21-10-0506 PMC9265156 35549314

[CR28] Hof L, Moreth T, Koch M, Liebisch T, Kurtz M, Tarnick J (2021). Long-term live imaging and multiscale analysis identify heterogeneity and core principles of epithelial organoid morphogenesis. BMC Biol.

[CR29] Hoffman DP, Shtengel G, Xu CS, Campbell KR, Freeman M, Wang L, et al (2020) Correlative three-dimensional super-resolution and block-face electron microscopy of whole vitreously frozen cells. Science 367(6475):eaaz5357 10.1126/science.aaz5357 PMC7339343 31949053

[CR30] Hoover EE, Squier JA (2013). Advances in multiphoton microscopy technology. Nat Photonics.

[CR31] Hopt A, Neher E (2001). Highly nonlinear photodamage in two-photon fluorescence microscopy. Biophys J.

[CR32] Horton NG, Wang K, Kobat D, Clark CG, Wise FW, Schaffer CB (2013). In vivo three-photon microscopy of subcortical structures within an intact mouse brain. Nat Photonics.

[CR33] Jakob PH, Kehrer J, Flood P, Wiegel C, Haselmann U, Meissner M (2016). A 3-D cell culture system to study epithelia functions using microcarriers. Cytotechnology.

[CR34] Ji N (2017). Adaptive optical fluorescence microscopy. Nat Methods.

[CR35] Ji N, Shroff H, Zhong H, Betzig E (2008). Advances in the speed and resolution of light microscopy. Curr Opin Neurobiol.

[CR36] Johnson C, Exell J, Lin Y, Aguilar J, Welsher KD (2021) Capturing the start point of the virus-cell interaction with high-speed 3D single-virus tracking. Nat Methods 5:11753 10.1038/s41592-022-01672-3 PMC10154077 36357694

[CR37] Keller PJ, Pampaloni F, Stelzer EHK (2006). Life sciences require the third dimension. Curr Opin Cell Biol.

[CR38] Keller PJ, Schmidt A, Wittbrodt J, Stelzer EHK (2008). Reconstruction of zebrafish early embryonic development by scanned light sheet microscopy. Science.

[CR39] Kner P, Chhun BB, Griffis ER, Winoto L, Gustafsson MGL (2009). Super-resolution video microscopy of live cells by structured illumination. Nat Methods.

[CR40] Kopek BG, Shtengel G, Xu CS, Clayton DA, Hess HF (2012). Correlative 3D superresolution fluorescence and electron microscopy reveal the relationship of mitochondrial nucleoids to membranes. Proc Natl Acad Sci USA.

[CR41] Krishna Inavalli VVG, Lenz MO, Butler C, Angibaud J, Compans B, Levet F (2019). A super-resolution platform for correlative live single-molecule imaging and STED microscopy. Nat Methods.

[CR42] Laissue PP, Alghamdi RA, Tomancak P, Reynaud EG, Shroff H (2017). Assessing phototoxicity in live fluorescence imaging. Nat Methods.

[CR43] LeCun Y, Bengio Y, Hinton G (2015). Deep learning. Nature.

[CR44] Ledig C, Theis L, Huszar F, Caballero J, Cunningham A, Acosta A et al (2017) Photo-realistic single image super-resolution using a generative adversarial network. In: IEEE Conference on Computer Vision and Pattern Recognition, pp 4681–4690

[CR45] Lelek M, Gyparaki MT, Beliu G, Schueder F, Griffié J, Manley S (2021). Single-molecule localization microscopy. Nat Rev Methods Prim.

[CR46] Li D, Shao L, Chen BC, Zhang X, Zhang M, Moses B (2015). Extended-resolution structured illumination imaging of endocytic and cytoskeletal dynamics. Science.

[CR47] Li Y, Su Y, Guo M, Han X, Liu J, Vishwasrao H (2022). Incorporating the image formation into deep learning improves network performance in deconvolution applications. Nat Methods.

[CR48] Liu T-L, Upadhyayula S, Milkie DE, Singh V, Wang K, Swinburne IA (2018). Observing the cell in its native state: imaging subcellular dynamics in multicellular organisms. Science.

[CR49] Mahecic D, Stepp WL, Zhang C, Griffie J, Weigert M, Manley S (2021). Event-driven acquisition for content-enriched microscopy. Nat Methods.

[CR50] Manley S, Gillette JM, Patterson GH, Shroff H, Hess HF, Betzig E (2008). High-density mapping of single-molecule trajectories with photoactivated localization microscopy. Nat Methods.

[CR51] McDole K, Guignard L, Amat F, Berger A, Malandain G, Royer LA (2018). In toto imaging and reconstruction of post-implantation mouse development at the single-cell level. Cell.

[CR52] Moyle MW, Barnes KM, Kuchroo M, Gonopolskiy A, Duncan LH, Sengupta T (2021). Structural and developmental principles of neuropil assembly in *C. elegans*. Nature.

[CR53] Nägerl UV, Willig KI, Hein B, Hell SW, Bonhoeffer T (2008). Live-cell imaging of dendritic spines by STED microscopy. Proc Natl Acad Sci USA.

[CR54] Ouyang W, Aristov A, Lelek M, Hao X, Zimmer C (2018). Deep learning massively accelerates super-resolution localization microscopy. Nat Biotechnol.

[CR55] Power RM, Huisken J (2017). A guide to light-sheet fluorescence microscopy for multiscale imaging. Nat Methods.

[CR56] Qiao C, Li D, Guo Y, Liu C, Jiang T, Dai Q (2021). Evaluation and development of deep neural networks for image super-resolution in optical microscopy. Nat Methods.

[CR57] Rodriguez C, Chen A, Rivera JA, Mohr MA, Liang Y, Natan RG (2021). An adaptive optics module for deep tissue multiphoton imaging in vivo. Nat Methods.

[CR58] Royer LA, Lemon WC, Chhetri RK, Wan Y, Coleman M, Myers EW (2016). Adaptive light-sheet microscopy for long-term, high-resolution imaging in live organisms. Nat Biotechnol.

[CR59] Rust MJ, Bates M, Zhuang X (2006). Sub-diffraction-limit imaging by stochastic optical reconstruction microscopy (STORM). Nat Methods.

[CR60] Santella A, Catena R, Kovacevic I, Shah P, Yu Z, Marquina-Solis J (2015). WormGUIDES: an interactive single cell developmental atlas and tool for collaborative multidimensional data exploration. BMC Bioinform.

[CR61] Sapoznik E, Chang B-J, Huh J, Ju RJ, Azarova EV, Pohlkamp T (2020). A versatile oblique plane microscope for large-scale and high-resolution imaging of subcellular dynamics. Elife.

[CR62] Sarder P, Nehorai A (2006). Deconvolution methods for 3-D fluorescence microscopy images. IEEE Signal Process Mag.

[CR63] Scherf N, Huisken J (2015). The smart and gentle microscope. Nat Biotech.

[CR64] Schermelleh L, Ferrand A, Huser T, Eggeling C, Sauer M, Biehlmaier O (2019). Super-resolution microscopy demystified. Nat Cell Biol.

[CR65] Schwarz C, Hentschke H, Butovas S, Haiss F, Stüttgen MC, Gerdjikov TV (2010). The head-fixed behaving rat—procedures and pitfalls. Somatosens Mot Res.

[CR66] Shao L, Kner P, Rego EH, Gustafsson MGL (2011). Super-resolution 3D microscopy of live whole cells using structured illumination. Nat Methods.

[CR67] Sharonov A, Hochstrasser RM (2006). Wide-field subdiffraction imaging by accumulated binding of diffusing probes. Proc Natl Acad Sci USA.

[CR68] Shroff H, Galbraith CG, Galbraith JA, Betzig E (2008). Live-cell photoactivated localization microscopy of nanoscale adhesion dynamics. Nat Methods.

[CR69] Stelzer EHK (2014). Light-sheet fluorescence microscopy for quantitative biology. Nat Methods.

[CR70] Streich L, Boffi JC, Wang L, Alhalaseh K, Barbieri M, Rehm R (2021). High-resolution structural and functional deep brain imaging using adaptive optics three-photon microscopy. Nat Methods.

[CR71] Sulston JE, Schierenberg E, White JG, Thomson JN (1983). The embryonic cell lineage of the nematode *Caenorhabditis* elegans. Dev Biol.

[CR72] Theer P, Denk W (2006). On the fundamental imaging-depth limit in two-photon microscopy. J Opt Soc Am A Opt Image Sci vis.

[CR73] Tomer R, Khairy K, Amat F, Keller PJ (2012). Quantitative high-speed imaging of entire developing embryos with simultaneous multiview light-sheet microscopy. Nat Methods.

[CR74] Tønnesen J, Krishna Inavalli VVG, Nägerl UV (2018). Super-resolution imaging of the extracellular space in living brain tissue. Cell.

[CR75] Vicidomini G, Bianchini P, Diaspro A (2018). STED super-resolved microscopy. Nat Methods.

[CR76] Voleti V, Patel KB, Li W, Campos CP, Bharadwaj S, Yu H (2019). Real-time volumetric microscopy of in vivo dynamics and large-scale samples with SCAPE 2.0. Nat Methods.

[CR77] Wäldchen S, Lehmann J, Klein T, van de Linde S, Sauer M (2015). Light-induced cell damage in live-cell super-resolution microscopys. Sci Rep.

[CR78] Walsh CL, Tafforeau P, Wagner WL, Jafree DJ, Bellier A, Werlein C (2021). Imaging intact human organs with local resolution of cellular structures using hierarchical phase-contrast tomography. Nat Methods.

[CR80] Wang K, Milkie DE, Saxena A, Engerer P, Misgeld T, Bronner ME (2014). Rapid adaptive optical recovery of optimal resolution over large volumes. Nat Methods.

[CR81] Wang K, Sun W, Richie CT, Harvey BK, Betzig E, Ji N (2015). Direct wavefront sensing for high-resolution in vivo imaging in scattering tissue. Nat Commun.

[CR82] Wang T, Ouzounov DG, Wu C, Horton NG, Zhang B, Wu C-H (2018). Three-photon imaging of mouse brain structure and function through the intact skull. Nat Methods.

[CR83] Wang X, Yu K, Wu S, Gu J, Liu Y, Dong C et al (2018b) ESRGAN: enhanced super-resolution generative adversarial networks. Comput Vision and Pattern Recognit. 10.48550/arXiv.1809.00219

[CR79] Wang H, Rivenson Y, Jin Y, Wei Z, Gao R, H, G., (2019). Deep learning enables cross-modality super-resolution in fluorescence microscopy. Nat Methods.

[CR84] Weigert M, Schmidt U, Boothe T, Muller A, Dibrov A, Jain A (2018). Content-aware image restoration: pushing the limits of fluorescence microscopy. Nat Methods.

[CR85] Westphal V, Rizzoli SO, Lauterbach MA, Kamin D, Jahn R, Hell SW (2008). Video-rate far-field optical nanoscopy dissects synaptic vesicle movement. Science.

[CR86] White JG, Southgate E, Thomson JN, Brenner S (1986). The structure of the nervous system of the nematode* Caenorhabditis elegans*. Phil Trans R Soc Lond Ser B.

[CR87] Winter PW, Shroff H (2014). Faster fluorescence microscopy: advances in high speed biological imaging. Curr Opin Chem Biol.

[CR88] Wu Y, Christensen R, Colon-Ramos D, Shroff H (2013). Advanced optical imaging techniques for neurodevelopment. Curr Opin Neurobiol.

[CR89] Wu Y, Ghitani A, Christensen R, Santella A, Du Z, Rondeau G (2011). Inverted selective plane illumination microscopy (iSPIM) enables coupled cell identity lineaging and neurodevelopmental imaging in* Caenorhabditis elegans*. Proc Natl Acad Sci USA.

[CR90] Wu Y, Han X, Su Y, Glidewell M, Daniels JS, Liu J (2021). Multiview confocal super-resolution microscopy. Nature.

[CR91] Wu Y, Kumar A, Smith C, Ardiel EL, Chandris P, Christensen R (2017). Reflective imaging improves spatiotemporal resolution and collection efficiency in light sheet microscopy. Nat Commun.

[CR92] Wu Y, Shroff H (2018). Faster, sharper, and deeper: structured illumination microscopy for biological imaging. Nat Methods.

[CR93] Wu Y, Wawrzusin P, Senseney J, Fischer RS, Christensen R, Santella A (2013). Spatially isotropic four-dimensional imaging with dual-view plane illumination microscopy. Nat Biotechnol.

[CR94] Yang B, Chen X, Wang Y, Feng S, Pessino V, Stuurman N (2019). Epi-illumination SPIM for volumetric imaging with high spatial-temporal resolution. Nat Methods.

[CR95] York AG, Chandris P, Nogare DD, Head J, Wawrzusin P, Fischer RS (2013). Instant super-resolution imaging in live cells and embryos via analog image processing. Nat Methods.

[CR96] York AG, Parekh SH, Dalle Nogare D, Fischer RS, Temprine K, Mione M (2012). Resolution doubling in live, multicellular organisms via multifocal structured illumination microscopy. Nat Methods.

[CR97] Zheng W, Wu Y, Winter PW, Fischer RS, Nogare DD, Hong A (2017). Adaptive optics improves multiphoton super-resolution imaging. Nat Methods.

